# Extracellular vesicles as nanocarriers in glioblastoma: implications for chemoresistance and immune evasion

**DOI:** 10.3389/fnmol.2026.1705115

**Published:** 2026-06-16

**Authors:** Faezeh Ebrahimi, Taha Rahavi Ezabadi, Hale Asghari, Shima Rajaeinejad, Sahar Jalili, Shokuh Aghamirzaei, Erfan Shahabinejad, Fateme Sadat Kouchakzade, Farid Abbaspour, Abbas Lalegani, Ali Pirsadeghi, Taral R. Lunavat

**Affiliations:** 1 Student Research Committee, Rafsanjan University of Medical Sciences, Rafsanjan, Iran; 2 USERN Office, Rafsanjan University of Medical Sciences, Rafsanjan, Iran; 3 Student Research Committee, Shahid Sadoughi University of Medical Sciences, Yazd, Iran; 4 School of Medicine, Shahid Sadoughi University of Medical Sciences, Yazd, Iran; 5 Student Research Committee, Department of Medicine, Iran University of Medical Sciences, Tehran, Iran; 6 Faculty of Medicine, Medical Branch of Tehran, Islamic Azad University, Tehran, Iran; 7 Department of Nursing, School of Nursing and Midwifery, Qazvin University of Medical Sciences, Qazvin, Iran; 8 Department of Internal Medicine, Tehran University of Medical Sciences, Tehran, Iran; 9 Student Research Committee, Afzalipour Faculty of Medicine, Kerman University of Medical Sciences, Kerman, Iran; 10 Department of International Education, I.M. Sechenov First Moscow State Medical University, Moscow, Russia; 11 School of Medicine, Isfahan University of Medical Sciences, Isfahan, Iran; 12 Student Research Committee, Ahvaz Jundishapur University of Medical Sciences, Ahvaz, Iran; 13 Section for Pharmacology and Pharmaceutical Biosciences, Department of Pharmacy, Faculty of Mathematics and Natural Sciences, University of Oslo, Oslo, Norway

**Keywords:** biomarkers, chemoresistance, drug efflux, extracellular vesicles, glioblastoma, immune evasion, therapeutic targets, tumor microenvironment

## Abstract

Glioblastoma (GBM), the most aggressive primary brain tumor, remains highly resistant to conventional therapies because of its pronounced intratumoral heterogeneity, adaptive plasticity, and complex tumor microenvironment. Increasing evidence indicates that extracellular vesicles (EVs) play central roles in mediating intercellular communication within GBM and contribute to multiple mechanisms associated with therapeutic resistance. This review critically examines the biogenesis, molecular cargo composition, and functional heterogeneity of GBM-derived EVs, with particular emphasis on their involvement in chemoresistance-related processes, including apoptosis evasion, DNA repair modulation, stemness maintenance, immune reprogramming, metabolic adaptation, and remodeling of the tumor microenvironment. EV-associated cargos, including microRNAs, long non-coding RNAs, circular RNAs, proteins, and lipids, appear to coordinately regulate interconnected resistance networks under therapeutic pressure rather than functioning through isolated pathways. Importantly, this review distinguishes between findings derived from *in vitro* systems, preclinical *in vivo* models, and patient-derived clinical evidence in order to better contextualize the current translational relevance of EV-based mechanisms. Emerging evidence further suggests that EV-mediated effects may be context-dependent and, in certain settings, may exert non-canonical or antiproliferative functions. In addition to their pathological roles, EVs are increasingly being investigated as potential biomarkers and therapeutic platforms for GBM management. However, substantial translational barriers remain, including EV heterogeneity, lack of isolation standardization, scalability limitations, cargo-loading inefficiency, regulatory challenges, and incomplete understanding of off-target and physiological effects. By integrating mechanistic insights with translational considerations, this review provides a balanced framework for understanding the dual and context-dependent roles of EVs in GBM progression and therapeutic resistance while highlighting critical knowledge gaps that must be addressed before clinical implementation.

## 1 Introduction

Glioblastoma is the most aggressive type of glioma, a tumor that grows from glial cells and accounts for approximately 80% of malignant brain tumors (Li J. et al., 2022). Diagnosing glioblastoma involves neurological examination, imaging techniques such as MRI, CT, and PET, alongside histopathological and molecular analyses (Dymova et al., 2021). Despite multimodal treatment strategies, including surgery, radiotherapy, and temozolomide (TMZ)-based chemotherapy, glioblastoma almost inevitably recurs, often with increased aggressiveness and therapy resistance (Musatova and Rubtsov, 2023).

Multiple mechanisms contribute to treatment failure in glioblastoma, including limited drug penetration due to the blood-brain barrier and extensive inter- and intratumoral heterogeneity (Dymova et al., 2021). This heterogeneity is driven by clonal evolution, glioma stem-like cell plasticity, drug efflux mechanisms, metabolic adaptation, tumor–microenvironment interactions, and enhanced DNA repair capacity, all of which contribute to chemoresistance (Yekula et al., 2021; Zhang et al., 2019). Recent studies have identified extracellular vesicles (EVs) as important mediators of intercellular communication in glioblastoma, facilitating the transfer of resistance-associated molecules between tumor cells and components of the tumor microenvironment (Familtseva et al., 2019). According to the Minimal Information for Studies of Extracellular Vesicles (MISEV) guidelines, the term “extracellular vesicles (EVs)” is recommended as an operational definition when specific biogenesis pathways cannot be definitively assigned (Sheta et al., 2023; Zhang et al., 2024a).

Extracellular vesicles are nanosized membrane-bound vesicles that carry a wide range of biomolecules, including nucleic acids, proteins, lipids, and metabolites, and play a key role in cell-to-cell communication (Garg et al., 2024). Through the transfer of this molecular cargo, EVs can influence tumor progression, immune evasion, and resistance to therapy, thereby contributing to the dynamic remodeling of the glioblastoma microenvironment (Babaei et al., 2024).

In particular, EV-mediated transfer of bioactive molecules such as microRNAs, long non-coding RNAs, proteins, and lipids has been associated with modulation of drug efflux pathways, regulation of apoptosis and DNA repair, promotion of stem-like phenotypes, and adaptation to therapeutic stress, all of which are implicated in the development of chemoresistance in glioblastoma (Gurung et al., 2021; Indira Chandran et al., 2024; Lunavat et al., 2023; Polónia et al., 2023).

Importantly, much of the current mechanistic understanding of EV-mediated chemoresistance and immune modulation in glioblastoma is derived from *in vitro* studies, glioma stem-like cell models, and preclinical systems, whereas direct clinical validation in patient-derived biofluids remains comparatively limited. In addition, the functional effects of GBM-derived EVs appear to be context-dependent and may vary according to tumor subtype, cellular origin, molecular cargo, and microenvironmental conditions, highlighting the need for cautious interpretation of their therapeutic and clinical relevance

## 2 Methods—literature search strategy

For this narrative review, we performed a structured literature search in PubMed, Scopus, Web of Science, and Google Scholar to identify studies investigating extracellular vesicles (EVs) in glioblastoma (GBM), with a particular focus on chemoresistance, immune evasion, and EV-based biomarkers or therapeutic strategies. The main search terms combined controlled vocabulary and free-text words related to “glioblastoma” or “GBM” and “extracellular vesicles,” “exosomes,” “microvesicles,” “nanocarriers,” “chemoresistance,” “drug resistance,” “immune evasion,” “tumor microenvironment,” and “liquid biopsy.” Searches were limited to English-language, peer-reviewed original and review articles published between January 2000 and October 2025. We additionally screened the reference lists of key primary papers and recent reviews on GBM-derived EVs to identify relevant studies not captured in the initial search. Articles were included if they reported experimental or clinical data on GBM EV biogenesis, cargo composition, functional effects on chemoresistance or immune modulation, or the use of GBM-derived EVs as biomarkers or therapeutic targets. Studies focusing exclusively on other brain tumors, non-vesicular secreted factors, or non-oncologic EV applications were excluded. EV terminology and reporting in this review follow current MISEV recommendations, using “extracellular vesicles (EVs)” as an operational umbrella term whenever specific biogenesis pathways cannot be unequivocally assigned.

## 3 Extracellular vesicles in glioblastoma biology and heterogeneity

### 3.1 Glioblastomas: heterogeneity and therapeutic challenges

Glioblastoma exhibits profound heterogeneity shaped by both intrinsic and extrinsic determinants, including genetically diverse clonal and subclonal neoplastic populations, glioma stem-like cells (GSCs), and tumor microenvironment (TME) components (Brosque and Friedmann-Morvinski, 2023; Indira Chandran et al., 2024). This multilayered heterogeneity is widely recognized as a key contributor to therapeutic resistance, adaptive tumor evolution, and variable clinical outcomes. However, it is important to note that the relative contribution of each of these components may vary depending on tumor subtype, disease stage, and experimental context, which complicates the interpretation of therapeutic responses.

### 3.2 Mechanisms of glioblastoma heterogeneity

Intra-tumoral heterogeneity arises primarily from intrinsic factors such as the cell of origin and accumulated genetic and epigenetic alterations that drive tumor progression (Puchalski et al., 2018). These intrinsic alterations generate a spectrum of cellular states and phenotypic traits, enabling glioma cells to undergo plastic transitions toward more aggressive and therapy-resistant phenotypes. At the same time, inter-tumoral heterogeneity is further shaped by extrinsic influences derived from the TME, including immune composition, stromal interactions, and hypoxic niches (Indira Chandran et al., 2024; Puchalski et al., 2018).

Comprehensive gene expression profiling has identified three principal molecular subtypes of glioblastoma: proneural (PN), mesenchymal (MES), and classical (CL). These subtypes are commonly associated with PDGFRA and IDH1 alterations (PN), EGFR amplification (CL), and NF1 loss (MES) (Liguori, 2024; Wang et al., 2017). Subtype-specific transcriptional programs contribute to differential therapeutic responses, reflecting variations in receptor tyrosine kinase signaling, tumor suppressor pathways, and metabolic regulation (Indira Chandran et al., 2024). However, it should be noted that these subtype classifications are derived primarily from bulk transcriptomic analyses and may not fully capture the cellular diversity observed at the single-cell level.

Epigenetic mechanisms, including chromatin remodeling, histone modification, and DNA methylation, play a central role in shaping dynamic gene expression patterns in GBM. DNA methylation patterns often parallel chromosomal abnormalities, as regions with copy number loss exhibit hypermethylation, whereas amplified regions such as chromosome seven demonstrate reduced methylation levels (Chaligne et al., 2021; Romani et al., 2018). Nevertheless, the causal relationship between these epigenetic alterations and therapy resistance remains incompletely understood.

Importantly, glioblastoma subtypes are not static, and tumor cells undergo dynamic interconversion between cellular states during progression and recurrence, driven by transcriptional and epigenetic plasticity in response to therapeutic and microenvironmental pressures. (Amirmahani et al., 2025; Wang et al., 2017). Spatially separated tumor regions frequently display distinct molecular subtypes and copy number alterations involving genes such as EGFR, PTEN, and PDGFR, underscoring the coexistence of multiple cellular lineages within a single tumor mass (Sottoriva et al., 2013). These observations highlight the limitations of static subtype classification systems in predicting treatment response.

Glioma stem-like cells (GSCs) are recognized as key contributors to GBM aggressiveness, owing to their capacity for self-renewal, multipotent differentiation, and marked cellular plasticity (Amirmahani et al., 2025; Gimple et al., 2019; Johnson et al., 2022). However, it should be noted that these subtype classifications are derived primarily from bulk transcriptomic analyses and may not fully capture the cellular diversity observed at the single-cell level, as recent single-cell studies have demonstrated that cancer cell states exist along a dynamic and continuous spectrum rather than as strictly discrete categories (Holton et al., 2024).

Therapeutic pressure further reshapes the glioblastoma landscape by altering cellular states and promoting the emergence of resistant clones. Single-cell RNA sequencing studies have revealed substantial inter-patient variability in immune cell composition within the TME, highlighting the combined impact of intrinsic tumor evolution and microenvironmental influences on disease heterogeneity (Abdelfattah et al., 2022; Indira Chandran et al., 2024). Despite these advances, the integration of single-cell and spatial transcriptomic findings into clinically actionable frameworks remains a significant challenge, particularly due to limitations in scalability, reproducibility, and direct clinical translation (Gulati et al., 2025).

### 3.3 Communication through EVs in the heterogeneity of glioblastoma

Glioblastoma heterogeneity is influenced, in part, by tumor–microenvironment interactions mediated by extracellular vesicles (EVs) (Yekula et al., 2020). Through autocrine and paracrine signaling, EVs facilitate bidirectional communication within the tumor microenvironment (TME), thereby contributing to dynamic tumor evolution. Oncogenic cargo such as EGFRvIII can be transferred via EVs, which have been associated with modulation of receptor tyrosine kinase signaling and may contribute to a more aggressive phenotype (Indira Chandran et al., 2024). In addition, EV-mediated transfer of microRNAs (miRNAs) has been implicated in the regulation of tumor cell behavior, including proliferation, invasion, and therapy response; however, these effects appear to be context-dependent and vary across experimental models (Godlewski et al., 2017; He et al., 2021). Furthermore, intratumoral exchange of EV-associated miRNAs between glioma stem-like cells (GSCs) and other tumor subpopulations has been shown to influence molecular and phenotypic diversity, potentially enhancing cellular plasticity within GBM (Godlewski et al., 2017; Indira Chandran et al., 2024).

### 3.4 Role of EVs in intercellular communication

Extracellular vesicles (EVs) contribute to intercellular communication by transferring bioactive cargo, including proteins, nucleic acids, lipids, and metabolites, to recipient cells under both physiological and pathological conditions (Gurung et al., 2021). EVs can interact with target cells through multiple mechanisms, including endocytosis, membrane fusion, and ligand–receptor interactions, although the relative contribution of each pathway may vary depending on cell type and context (Kwok et al., 2021).

These interactions enable EVs to influence intracellular signaling pathways and cellular functions, including gene expression and metabolic activity. Consequently, EV-mediated communication has been implicated in the modulation of both local and systemic processes associated with tumor progression; however, these effects remain highly context-dependent and are not yet fully understood (Gurung et al., 2021).

### 3.5 EV formation in cancer cells

Extracellular vesicles are nanoscale membrane-bound vesicles released by a wide range of normal and tumor cells and can carry diverse bioactive molecules, including RNAs, proteins, and lipids (Zhang et al., 2024b). Extracellular vesicles are broadly classified into exosomes, microvesicles, and apoptotic bodies based on their biogenesis and size; however, in many experimental contexts, a clear distinction between these subtypes remains challenging due to overlapping characteristics and limitations in isolation techniques (Chen and Yang, 2024). Tumor cells, including glioblastoma cells, generally exhibit increased EV secretion compared to non-malignant cells (Dai et al., 2024). In the brain, both neurons and glial cells release EVs, and *in vitro* studies using cultured glioma cells suggest that a single glioma cell can release up to ∼10,000 EVs within 48 h (Whitehead et al., 2019). These vesicles are capable of crossing the blood-brain barrier in both directions and can be detected in peripheral biofluids, supporting their potential as circulating biomarkers (Jafari et al., 2020).

Glioblastoma -derived EV cargo reflects both tumor-intrinsic characteristics and microenvironmental influences. These vesicles contain a variety of immunomodulatory molecules, such as IL-10, TGF-β, heat shock proteins, and PD-L1 (Del Bene et al., 2022; Ricklefs et al., 2018), and their molecular composition may mirror the originating tumor tissue, providing insights into tumor status and progression (Hallal et al., 2019). Functionally, EVs mediate intercellular communication by modulating gene expression through receptor–ligand interactions at the cell surface and by delivering bioactive cargo to nearby or distant cells via cerebrospinal fluid and the bloodstream (Del Bene et al., 2022). However, variability in EV isolation, characterization methods, and lack of standardized protocols remain significant limitations, which may affect reproducibility and interpretation of findings across studies (Ragni, 2025).

Importantly, glioma cell heterogeneity is reflected in EV composition. Distinct glioma stem cell (GSC) subtypes release EVs with different molecular profiles. For example, EVs derived from mesenchymal GSCs are enriched in tetraspanins such as CD9, CD63, and CD81, whereas proneural GSC-derived EVs show reduced expression of these canonical markers (Spinelli et al., 2018). This subtype-dependent variation extends to EV biogenesis pathways, cargo composition, and functional properties, highlighting the role of EVs in GBM heterogeneity (Indira Chandran et al., 2024) Furthermore, selective packaging of signaling molecules into EVs enables GSCs to influence surrounding cells and reshape the tumor microenvironment. For instance, pro-angiogenic factors such as VEGF-A are transported within EVs and can enhance endothelial permeability and angiogenesis, while signaling proteins such as Notch1 can be transferred to recipient glioma cells, promoting stemness and tumorigenicity (Sun et al., 2020; Treps et al., 2017). These findings underscore the role of EV-mediated cargo transfer in tumor progression, intercellular reprogramming, and maintenance of the cancer stem cell niche.

Hypoxic regions within GBM further influence EV production and composition. Hypoxia induces genomic and secretory alterations that enhance EV release and modify their cargo, promoting angiogenesis, pericyte recruitment, and tumor cell proliferation. These hypoxia-associated EVs contribute additional layers of intratumoral heterogeneity and facilitate tumor progression and expansion (Indira Chandran et al., 2024). These findings further support the concept that EVs function as dynamic mediators of phenotypic plasticity and intercellular communication within the heterogeneous GBM microenvironment.

### 3.6 EV cargo in GBM

**FIGURE 1:**
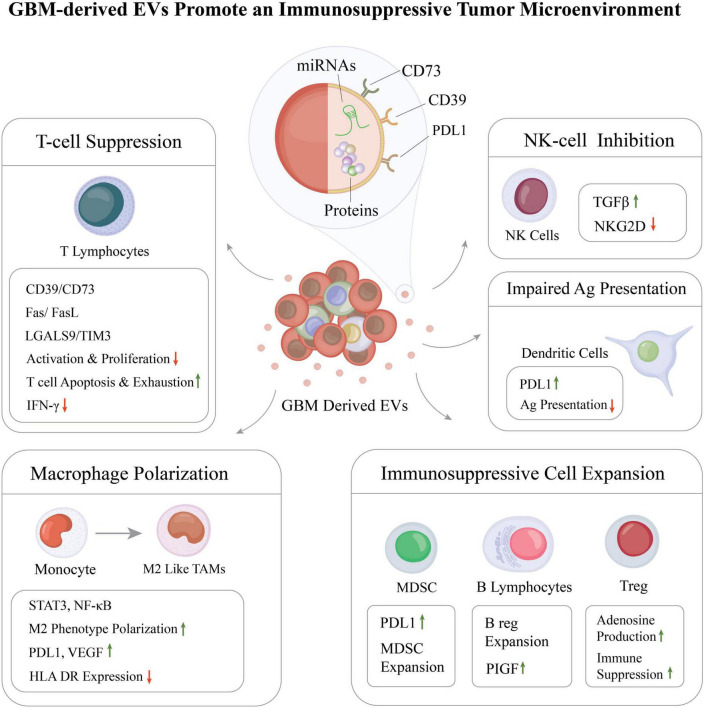
Conceptual overview of glioblastoma-derived extracellular vesicle (GBM-EV)-mediated immune suppression within the tumor microenvironment. GBM-EVs modulate both innate and adaptive immune responses through the transfer of immunoregulatory proteins, miRNAs, and surface-associated molecules. EV-mediated signaling contributes to T-cell exhaustion and apoptosis, suppression of NK-cell cytotoxicity, impaired dendritic-cell antigen presentation, expansion of immunosuppressive MDSC, Treg, and Breg populations, and polarization of monocyte/macrophage populations toward an M2-like immunosuppressive phenotype. Representative mechanisms are illustrated to summarize the major immunosuppressive pathways associated with GBM-EVs rather than provide an exhaustive molecular interaction map.

Glioblastoma-derived extracellular vesicles (GBM-EVs) carry a diverse repertoire of bioactive cargo, including proteins, DNA, RNA, lipids, and metabolites, which have been implicated in tumor progression, therapeutic resistance, and microenvironmental remodeling across multiple experimental systems. Rather than acting through isolated mechanisms, EV cargo may influence interconnected biological processes such as apoptosis regulation, DNA repair, oncogenic signaling, immune modulation, and tumor–microenvironment communication. Collectively, GBM-derived EVs contribute to tumor progression through coordinated modulation of immune suppression, therapeutic resistance, angiogenesis, stemness maintenance, and tumor–microenvironment communication, with immune remodeling representing a major EV-mediated mechanism within the GBM microenvironment (Figure 1). However, most mechanistic evidence remains derived from *in vitro* and preclinical glioma models, whereas direct clinical validation of EV-cargo function remains comparatively limited. In addition to their functional roles, EVs have also emerged as promising candidates for disease diagnosis, staging, grading, and early monitoring in malignancies (Yang et al., 2024).

#### 3.6.1 Glioblastoma-EV RNAs in chemoresistance

**FIGURE 2:**
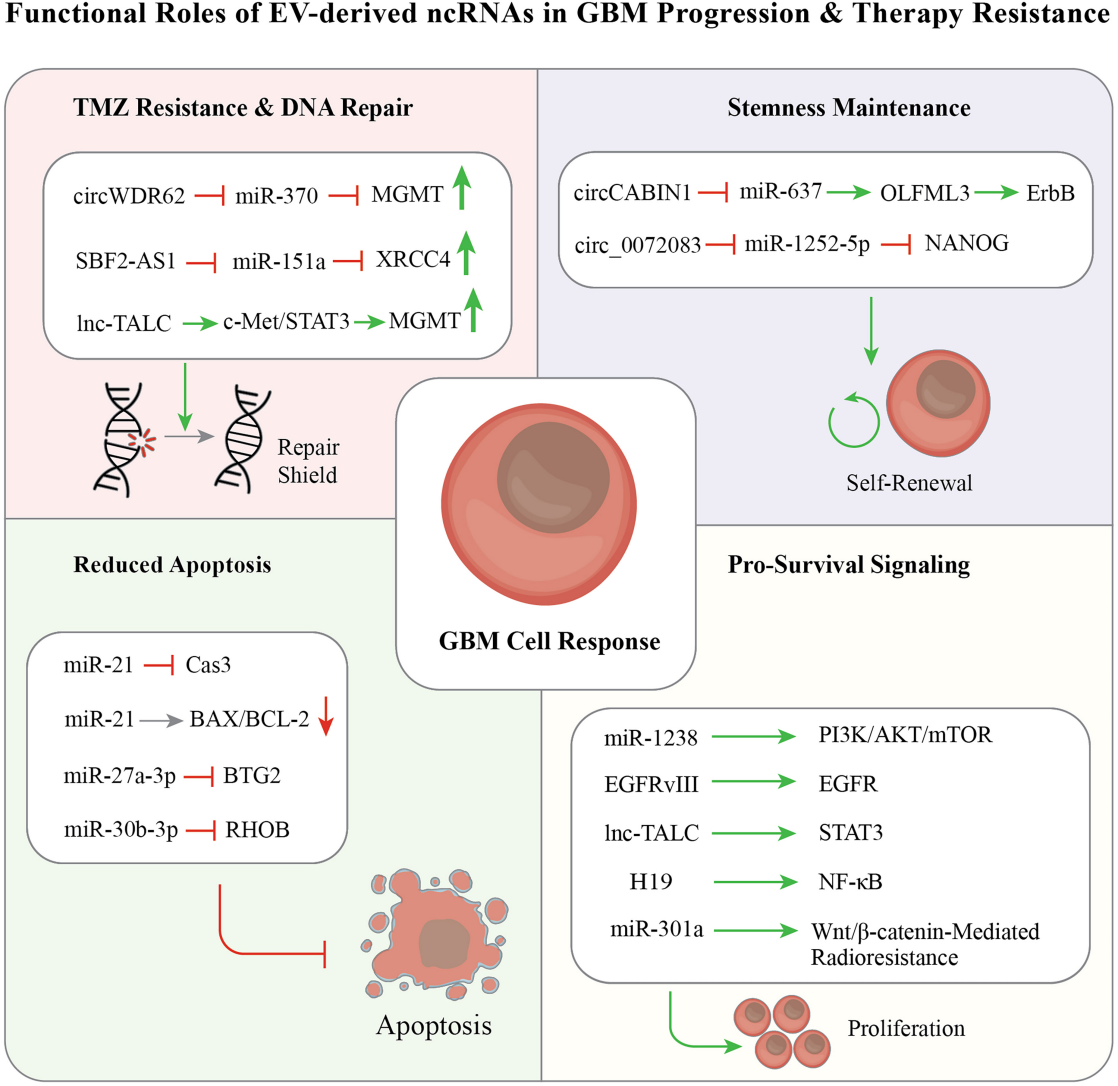
Conceptual overview of EV-associated non-coding RNA-mediated mechanisms involved in glioblastoma progression and therapy resistance. EV-derived miRNAs, lncRNAs, and circRNAs modulate key resistance-associated processes, including DNA repair, apoptosis suppression, stemness maintenance, activation of PI3K/AKT/mTOR and WNT/β-catenin signaling pathways, temozolomide resistance, and radioresistance. Representative ncRNA-mediated interactions involved in MGMT regulation, XRCC4-associated DNA repair, and pro-survival signaling are illustrated. The Figure is intended as a conceptual summary rather than a comprehensive molecular pathway map.

Collectively, EV-associated ncRNAs contribute to GBM progression and therapy resistance through several interconnected mechanisms, including apoptosis suppression, DNA repair modulation, stemness maintenance, activation of survival signaling pathways, and resistance to chemotherapy and radiotherapy (Figure 2).

##### 3.6.1.1 MicroRNAs (miRs)

Non-coding RNAs carried by glioblastoma-derived EVs regulate multiple pathways associated with drug response, tumor survival, and adaptive resistance mechanisms (Yang et al., 2024). Among these, microRNAs (miRs) are key post-transcriptional regulators that can influence cell cycle control, drug metabolism, apoptosis, DNA repair, and major oncogenic signaling pathways involved in TMZ response (Movahedpour et al., 2021; Palizkaran Yazdi et al., 2024). Temozolomide (TMZ), the most widely used first-line chemotherapeutic agent for GBM, has been reported to affect miRNA-associated resistance pathways.(Movahedpour et al., 2021). For example, miR-21 is upregulated in TMZ-resistant GBM cells generated by chronic TMZ exposure, and inhibition of miR-21 increases apoptosis and enhances TMZ-induced cytotoxicity *in vitro* (Wong et al., 2012). Mechanistically, miR-21-mediated resistance has been linked to reduced caspase-3 activity and a decreased BAX/BCL-2 ratio in GBM cell models (Wong et al., 2012). In contrast, some miRNAs appear to enhance TMZ sensitivity rather than resistance. MiR-139 directly targets the anti-apoptotic protein Mcl-1, and its overexpression suppresses proliferation and potentiates TMZ-induced apoptosis in glioma cell models, with additional support from xenograft experiments and inverse miR-139/Mcl-1 correlation in glioma tissues (Li et al., 2013). Similarly, miR-143 acts as a tumor-suppressive miRNA by directly targeting N-RAS and inhibiting downstream PI3K/AKT, MAPK/ERK, and NF-κB-related signaling; restoration of miR-143 enhances TMZ-induced apoptosis and reduces tumor growth *in vitro* and *in vivo* (Wang et al., 2014).

Beyond apoptosis-related mechanisms, EV-associated miRNAs have also been implicated in regulating major oncogenic signaling cascades and resistance-associated pathways. For example, miR-1238 is enriched in EVs derived from TMZ-resistant GBM cells. It can be transferred to TMZ-sensitive recipient cells, where it has been reported to promote resistance-related phenotypes. Mechanistically, this effect has been linked to modulation of the CAV1/EGFR signaling axis, resulting in activation of downstream EGFR–PI3K–Akt–mTOR signaling pathways in GBM models, primarily supported by *in vitro* and preclinical evidence (Yin et al., 2019).

Consistent with this broader regulatory role, exosomal miRNAs have been suggested to modulate regulatory proteins and their corresponding genes, or to influence multiple signaling pathways involved in drug resistance in GBM (Movahedpour et al., 2021). However, these effects are often context-dependent and largely supported by experimental models rather than clinical validation.

For instance, exosomal miR-221 has been shown to promote glioma progression and TMZ resistance by directly targeting the DNM3 (dynamin-3) gene. Functional studies demonstrate that exosome-mediated transfer of miR-221 enhances proliferation, migration, and drug resistance in GBM cell models, supporting its role as a pro-oncogenic EV-associated miRNA (Yang et al., 2017).

Similarly, exosomal miR-25-3p is upregulated in TMZ-resistant GBM cells and can be transferred to sensitive cells, where it promotes proliferation and resistance by targeting FBXW7. This results in increased expression of oncogenic proteins such as c-Myc and cyclin E, with supporting evidence from both *in vitro* experiments, xenograft models, and patient serum samples (Wang J. et al., 2021).

Importantly, EV-mediated miRNA transfer does not universally promote resistance and may also enhance therapeutic sensitivity. For example, miR-151a has been identified as a chemosensitizing miRNA whose reduced expression contributes to TMZ resistance. Restoration of exosomal miR-151a suppresses XRCC4-mediated DNA repair, thereby increasing DNA damage and enhancing TMZ-induced apoptosis in GBM models, with evidence from *in vitro*, *in vivo*, and patient-derived samples (Zeng et al., 2018).

Extracellular vesicle-associated miRNAs have additionally been implicated in radiation response and hypoxia-driven resistance mechanisms. MiR-301a released by hypoxic GBM cells can be transferred to normoxic-cultured cells, suppressing TCEAL7 and activating Wnt/β-catenin signaling, with potential relevance to radiation sensitivity and treatment effectiveness *in vitro* hypoxic GBM models (Yue et al., 2019). In addition, several EV miRNA cargoes (miR-144-4p, miR-320e, miR-155-5p, miR-363-3p, miR-16-5p, miR-23a-3p, miR-495-3p, and miR-520f-3p) have been associated with radiation resistance in GB-derived EVs *in vitro* (Ma et al., 2022).

Furthermore, EVs derived from hypoxic glioma stem-like cells (GSCs) exert a stronger effect on GBM chemoresistance compared to normoxic EVs. Mechanistically, miR-30b-3p is transcriptionally induced by HIF1α and STAT3, and its interaction with hnRNPA2B1 facilitates selective EV loading and transfer. EV-associated miR-30b-3p directly targets RHOB, leading to reduced apoptosis and enhanced proliferation in both *in vitro* systems and xenograft models, highlighting its role in hypoxia-mediated TMZ resistance (Yin et al., 2021). Similarly, exosomal miR-27a-3p promotes TMZ resistance by targeting BTG2, a tumor suppressor gene involved in cell cycle regulation and apoptosis. Functional studies demonstrate that EV-mediated delivery of miR-27a-3p enhances proliferation, reduces apoptosis, and increases resistance to TMZ in GBM cells, with validation in both *in vitro* experiments and xenograft mouse models (Chen et al., 2022).

Exosomal long non-coding RNAs (lncRNAs) have emerged as important regulators of therapeutic resistance in glioblastoma, influencing multiple treatment modalities including chemotherapy, targeted therapy, immunotherapy, and hormone therapy (De Los Santos et al., 2019). Rather than acting through isolated mechanisms, accumulating evidence suggests that lncRNAs contribute to TMZ resistance through several interconnected functional axes, including (i) regulation of DNA damage repair, (ii) modulation of proliferation and epithelial–mesenchymal transition (EMT), (iii) activation of survival and inflammatory signaling pathways, and (iv) remodeling of the tumor microenvironment (TME) and immune responses (Figure 2).

A major mechanism involves regulating DNA repair pathways. For example, lncRNA SBF2-AS1 functions as a competitive endogenous RNA (ceRNA) for miR-151a-3p, thereby relieving repression of XRCC4 and promoting DNA double-strand break (DSB) repair in GBM cells. Functionally, inhibition of SBF2-AS1 enhances TMZ sensitivity in resistant GBM models (*in vitro* evidence), supporting its role in DNA repair–mediated chemoresistance (Zhang et al., 2019).

In addition, several lncRNAs contribute to resistance by promoting proliferation, EMT, and oncogenic signaling. LncRNA HOTAIR is markedly upregulated in TMZ-resistant GBM cells and regulates tumor progression through multiple axes, including the miR-519a-3p/RRM1 pathway and miR-526b-3p/EVA1 signaling. These mechanisms promote proliferation, invasion, and EMT, with supporting evidence from *in vitro* studies, xenograft models, and detection in patient tissues and serum-derived EVs (Wang R. et al., 2022; Yuan et al., 2020).

Long non-coding RNAs also modulate survival signaling and stress-response pathways. For instance, lncRNA H19 is induced under oxidative stress conditions. It promotes TMZ resistance via activation of the NF-κB signaling pathway, thereby enhancing tumor cell survival and reducing apoptosis (*in vitro* evidence) (Duan et al., 2018). Similarly, MALAT1 contributes to chemoresistance by promoting multidrug resistance (MDR)-associated gene expression and facilitating EMT in GBM cells, further supporting its role in resistance-associated signaling networks (Li H. et al., 2017).

Beyond tumor-intrinsic mechanisms, exosomal lncRNAs play critical roles in reshaping the tumor microenvironment. LncRNA SNHG15 regulates the miR-627-5p/CDK6 axis, promoting proliferation, angiogenesis, and immunosuppressive microenvironmental changes. Notably, SNHG15 silencing restores TMZ sensitivity and reduces M2 polarization of glioma-associated microglia *in vitro* and in co-culture systems, highlighting its role in TME-mediated resistance (Li et al., 2019).

Similarly, ADAMTS9-AS2 has been reported to promote TMZ resistance through activation of the FUS/MDM2 signaling axis, thereby enhancing tumor cell survival in GBM models (*in vitro* evidence) (Yan et al., 2019).

Another clinically relevant lncRNA, lnc-TALC, originally identified in recurrent GBM, promotes TMZ resistance by modulating the c-Met signaling pathway. Mechanistically, lnc-TALC acts by sequestering miR-20b-3p and activating the STAT3/p300 complex, leading to upregulation of MGMT expression. Importantly, lnc-TALC can be packaged into exosomes and transferred to microglial cells, where it promotes M2 polarization and secretion of complement components, ultimately enhancing DNA repair and resistance. These findings are supported by preclinical models, including orthotopic tumors and *ex vivo* analyses, while direct clinical validation remains limited (Li et al., 2021).

Despite these advances, it is important to note that the majority of mechanistic insights regarding lncRNA-mediated EV functions in GBM are derived from *in vitro* systems and preclinical models. In addition, variability in EV isolation methods and the difficulty in distinguishing EV-mediated effects from intracellular lncRNA functions remain important limitations, which should be considered when interpreting these findings.

##### 3.6.1.3 Other classes of ncRNA

Circular RNAs (circRNAs) have emerged as important components of extracellular vesicles (EVs) that contribute to temozolomide (TMZ) resistance in glioblastoma through multiple coordinated mechanisms. Beyond their classical role as microRNA sponges, EV-associated circRNAs regulate interconnected processes including stemness maintenance, DNA repair, oncogenic signaling, and metabolic adaptation (Hua et al., 2019, 2020).

For example, circ_0072083 has been reported to regulate TMZ response through multiple miRNA-mediated mechanisms. Downregulation of circ_0072083 sensitizes glioma cells to TMZ via modulation of the miR-1294 axis (*in vitro* evidence) (Ding et al., 2021). Conversely, exosomal circ_0072083 can promote TMZ resistance by sponging miR-1252-5p, leading to increased NANOG expression and enhanced stemness properties in glioma cells, with supporting evidence from *in vitro* and xenograft models (Sun et al., 2020).

This circRNA is enriched in exosomes released from TMZ-resistant cells, enabling transfer of resistance phenotypes to recipient cells. Similarly, circ_0043949 has been identified as significantly upregulated in secondary TMZ-resistant GBM tissues, where it potentially functions through interaction with multiple miRNAs, including miR-7161-3p, miR-6783-3p, and miR-140-3p, suggesting a complex regulatory network underlying resistance (Zhao et al., 2020). In addition to ceRNA activity, circRNAs critically regulate glioma stemness and oncogenic signaling pathways. Exosomal circCABIN1 promotes TMZ resistance by sponging miR-637, resulting in upregulation of OLFML3 and activation of the ErbB signaling pathway, thereby enhancing glioma stem cell (GSC) self-renewal and resistance *in vitro* and in orthotopic *in vivo* models (Liu et al., 2023). Consistently, exosomal circ-HIPK3 contributes to TMZ resistance and tumor progression by modulating the miR-421/ZIC5 axis, promoting proliferation, invasion, and survival of glioma cells (*in vitro* evidence) (Han et al., 2021). Another key mechanism involves direct regulation of canonical chemoresistance pathways. Exosomal circWDR62 enhances TMZ resistance by sponging miR-370-3p, leading to upregulation of MGMT, a well-established mediator of TMZ resistance. Importantly, circWDR62 can be transferred via exosomes from resistant to sensitive cells, conferring resistance and promoting malignant phenotypes (*in vitro* evidence) (Geng et al., 2022).

Collectively, these findings indicate that EV-associated circRNAs function as central regulatory hubs integrating stemness, signaling, and DNA repair mechanisms to drive TMZ resistance.

However, several limitations should be considered. First, most of studies rely on *in vitro* systems and preclinical models, with limited validation in large patient cohorts. Second, many circRNA functions are inferred from predicted miRNA interactions rather than fully validated molecular networks. Finally, variability in EV isolation and characterization methods introduces challenges in reproducibility and interpretation of EV-mediated circRNA functions. Collectively, these studies demonstrate that EV-associated non-coding RNAs contribute to glioblastoma progression and therapeutic resistance through multiple interconnected mechanisms, including regulation of apoptosis, DNA repair, stemness, EMT, immune modulation, and oncogenic signaling pathways. A summary of the major EV-associated ncRNAs implicated in TMZ resistance and GBM progression is provided in Table 1.

**TABLE 1:** EV-associated non-coding RNAs involved in glioblastoma chemoresistance and tumor progression.

RNA types	Functional axis	Molecular target	Mechanism	Evidence level	EV origin	References
miR-151a	DNA repair/apoptosis	XRCC4	Loss of exosomal miR-151a enhances XRCC4-mediated DNA repair and promotes TMZ resistance; restoration of miR-151a suppresses XRCC4, increases DNA damage, and enhances TMZ-induced apoptosis in recipient GBM cells	*In vitro* + *in vivo* (xenograft) + clinical (CSF/serum EVs)	Exosomes derived from TMZ-resistant GBM cells act on TMZ-sensitive GBM cells	Zeng et al., 2018
miR-1238	Survival/drug resistance	CAV1	Exosomal miR-1238 suppresses CAV1, leading to activation of EGFR–PI3K/AKT/mTOR signaling and reduced apoptosis in recipient GBM cells	*In vitro* + *in vivo* (xenograft) + clinical (serum-derived exosomes)	TMZ-resistant GBM cell-derived exosomes	Yin et al., 2019
miR-27a-3p	Survival/drug resistance	BTG2 (tumor suppressor gene)	Exosomal miR-27a-3p targets and downregulates BTG2, promoting TMZ resistance	*In vitro* + *in vivo* (xenograft)	TMZ-resistant GBM cells/TMZ-sensitive GBM cells	Chen et al., 2022
miR-25-3p	Proliferation/drug resistance	FBXW7 (tumor suppressor gene)	Exosomal miR-25-3p downregulates FBXW7, leading to increased c-Myc and cyclin E expression and promoting TMZ resistance	*In vitro* + *in vivo* (xenograft) + clinical (serum EVs)	TMZ-resistant GBM cells/TMZ-sensitive GBM cells	Wang J. et al., 2021
MALAT1	EMT/drug resistance	MDR-related genes (MDR1, MRP5, LRP1)/EMT-related proteins (via ZEB1)	MALAT1 overexpression promotes TMZ resistance by upregulating MDR genes and inducing EMT through ZEB1 signaling	*In vitro*	TMZ-resistant GBM cells/TMZ-sensitive GBM cells	Li H. et al., 2017
ADAMTS9-AS2	Survival/drug resistance	FUS/MDM2/p53 axis	ADAMTS9-AS2 directly binds FUS and inhibits MDM2-mediated K48 ubiquitination, stabilizing FUS and promoting TMZ resistance	*In vitro* + clinical (patient tissues)	TMZ-resistant GBM cells/TMZ-sensitive GBM cells	Yan et al., 2019
HOTAIR	Proliferation/EMT/drug resistance	miR-519a-3p/RRM1	Exosomal HOTAIR acts as a ceRNA sponge for miR-519a-3p, leading to upregulation of RRM1 and promoting proliferation, EMT, and TMZ resistance	*In vitro* + *in vivo*	Resistant GBM cells/sensitive GBM cells	Yuan et al., 2020
circCABIN1	TMZ resistance/stemness)	miR-637/OLFML3	Acts as a ceRNA (miRNA sponge) for miR-637 → releases inhibition of OLFML3 → activates ErbB signaling → promotes stemness and TMZ resistance	*In vitro* + *in vivo* (GBM cell lines, tumorsphere assays, apoptosis assays, xenograft mouse model)	Glioblastoma cell–derived exosomes (TMZ-resistant GBM cells)	Zhao et al., 2020
circ-HIPK3	TMZ resistance/tumor progression	ZIC5 (via miR-421)	Upregulation of exosomal circ-HIPK3 sponges miR-421, leading to increased ZIC5 expression, which promotes cell proliferation, invasion, inhibits apoptosis, and enhances TMZ resistance in glioma cells	*In vitro* + *in vivo* (xenograft) + clinical samples (serum exosomes)	Exosomes derived from TMZ-resistant GBM cells acting on TMZ-sensitive GBM cells	Han et al., 2021
circWDR62	DNA repair/tumor progression/TMZ resistance	MGMT (via miR-370-3p)	Upregulation of exosomal circWDR62 sponges miR-370-3p, resulting in increased MGMT expression, which enhances DNA repair capacity, promotes proliferation, migration, invasion, and induces TMZ resistance in glioma cells	*In vitro* + *in vivo* (xenograft) + clinical (serum/tissue exosomes)	Exosomes derived from TMZ-resistant GBM cells were transferred to TMZ-sensitive GBM cells	Geng et al., 2022
circ_0072083	Stemness/TMZ resistance	miR-1252 (downstream targets related to resistance)	Upregulation of exosomal circ_0072083 promotes TMZ resistance by sponging miR-1252; conversely, its downregulation enhances TMZ sensitivity by inhibiting resistance-related signaling pathways	*In vitro* + *in vivo*	Exosomes derived from TMZ-resistant GBM cells act on recipient glioma cells	Ding et al., 2021
miR-221	Tumor progression/migration/TMZ resistance	DNM3 (dynamin-3)	Exosomal miR-221 directly targets and suppresses DNM3, promoting glioma cell proliferation, migration, anti-apoptotic activity, tumor growth, and TMZ resistance; inhibition of miR-221 reverses these effects and sensitizes cells to TMZ	*In vitro* + *in vivo* (xenograft) + clinical samples (serum exosomes/tissues)	U87MG glioma cell-derived exosomes acting on SHG-44 glioma cells	Yang et al., 2017
H19	Oxidative stress/NF-κB signaling/TMZ resistance	NF-κB signaling pathway	Oxidative stress-induced lncRNA H19 activates NF-κB signaling, thereby promoting TMZ tolerance and resistance in glioma cells	*In vitro*	TMZ-resistant glioma cells/other glioma cells	Jiang et al., 2016
SNHG15	Cell cycle/TMZ sensitivity	miR-627-5p/CDK6 axis	Silencing of SNHG15 suppresses the miR-627-5p/CDK6 axis, resulting in enhanced TMZ sensitivity in GBM cells	*In vitro*	GBM cells/microglial cells	Li et al., 2019
miR-30b-3p	Cell proliferation/anti-apoptosis/TMZ resistance	RHOB	EV-packaged miR-30b-3p directly targets RHOB, reducing apoptosis and promoting proliferation of recipient GBM cells, thereby enhancing TMZ resistance	*In vitro*	Hypoxic glioma stem-like cells/GBM cells	Yin et al., 2021
SBF2-AS1	DNA repair/chemoresistance	miR-151a-3p/XRCC4 axis	SBF2-AS1 acts as a ceRNA for miR-151a-3p, leading to disinhibition of XRCC4, which enhances DNA double-strand break repair and promotes TMZ resistance in GBM cells	*In vitro* + *in vivo* (xenograft) + clinical samples (serum exosomes/tissues)	TMZ-resistant GBM cell-derived exosomes acting on chemoresponsive GBM cells	Zhang et al., 2019
circCABIN1	Stemness/ErbB signaling/TMZ resistance	miR-637/OLFML3 axis	circCABIN1 regulates OLFML3 expression by sponging miR-637; OLFML3 overexpression activates ErbB downstream signaling, promoting stemness reprogramming and TMZ resistance	*In vitro* + *in vivo* (orthotopic xenograft) + clinical samples	TMZ-resistant GBM cell-derived exosomes	Liu et al., 2023
lnc-TALC	TMZ resistance/DNA repair/MGMT regulation	miR-20b-3p/MGMT/Stat3 axis	Exosomal lnc-TALC sponges miR-20b-3p, activates the Stat3 pathway, and increases MGMT expression, thereby promoting TMZ resistance and potentially facilitating resistance spread within the GBM microenvironment	*In vitro* + *in vivo* (xenograft) + clinical samples (GBM patient serum/plasma exosomes)	GBM cells/tumor microenvironment-derived exosomes	Li et al., 2021
miR-138	Immune reprogramming/anti-tumor immunity	TGF-β (tumor), M2-like TAMs	Engineered EV-delivered miR-138 repolarizes TAMs from an M2-like to M1-like phenotype, suppresses TGF-β signaling, increases IL-1β and CD8+ T-cell infiltration, and inhibits GBM progression	*In vitro* + *in vivo* (orthotopic/syngeneic GBM models)	Engineered trypsinized EVs targeting GBM cells and TAMs	Nguyen et al., 2025

EVs, extracellular vesicles; GBM, glioblastoma; TMZ, temozolomide; EMT, epithelial–mesenchymal transition; TAMs, tumor-associated macrophages; MGMT, O6-methylguanine-DNA methyltransferase; MDR, multidrug resistance; ceRNA, competing endogenous RNA; GSCs, glioma stem-like cells; CSF, cerebrospinal fluid; EGFR, epidermal growth factor receptor; PI3K, phosphoinositide 3-kinase; AKT, protein kinase B; mTOR, mammalian target of rapamycin; NF-κB, nuclear factor kappa B.

#### 3.6.2 Protein components from glioblastoma-EVs

Glioblastoma-derived extracellular vesicles (GBM-EVs) carry a diverse range of proteins that contribute to tumor–host interactions, immune modulation, angiogenesis, and therapy resistance. Notably, their functional impact appears to be context-dependent. In certain experimental settings, glioma-derived EVs have been reported to reduce tumor growth and Ki-67 expression in a C6 rat glioma model, suggesting potential antiproliferative and immune-modulatory effects under specific conditions (Scholl et al., 2020). However, most evidence supports a predominantly tumor-promoting role of GBM-EVs.

Glioblastoma-derived extracellular vesicles contribute to immune evasion through the transfer of immunosuppressive mediators such as galectin-9, indoleamine 2,3-dioxygenase (IDO), and transforming growth factor-beta (TGF-β), which collectively suppress anti-tumor immune responses (Lunavat et al., 2023b). This highlights the role of EVs in reshaping the tumor microenvironment toward an immunosuppressive state.

In addition to immune modulation, GBM-EVs transport proteins associated with angiogenesis and tumor aggressiveness. These include vascular endothelial growth factor (VEGF), matrix metalloproteinase 9 (MMP9), epidermal growth factor receptor (EGFR), platelet-derived growth factor receptor (PDGFR), and other pro-angiogenic mediators such as plasminogen activator, CXCR4, TGF-β1, and multiple proteases (Ngo and Harley, 2019; Quezada et al., 2018). Rather than acting independently, these factors operate within coordinated signaling networks that promote vascular remodeling, extracellular matrix degradation, and tumor progression.

Proteomic analyses further reveal enrichment of proteins involved in apoptosis resistance and cell adhesion, including heat shock protein 27 (HSP27) and CD44 (Szatanek and Baj-Krzyworzeka, 2021). Importantly, CD44-positive EVs have been implicated in enhancing tumor cell migration and endothelial tube formation, linking EV-mediated protein transfer to both invasive behavior and angiogenesis. This is consistent with the broader role of CD44 in facilitating tumor dissemination and microenvironmental interactions (Szatanek and Baj-Krzyworzeka, 2021).

Surface markers such as CD44 and CD133 are increasingly recognized not only as indicators of tumor aggressiveness but also as potential biomarkers associated with chemoresistance and disease progression (Quezada et al., 2018). For instance, lactate-induced upregulation of CD44 has been shown to enhance the release of CD44-enriched EVs, which in turn promote glioma cell migration and endothelial tube formation, while also enabling their detection in blood and cerebrospinal fluid as part of liquid biopsy approaches (Thakur et al., 2021).

Pro-angiogenic signaling is further intensified under hypoxic conditions. GBM-derived EVs exhibit increased levels of monocarboxylate transporter 1 (MCT1) and its associated protein CD147, both of which are implicated in metabolic adaptation, extracellular matrix remodeling, and enhanced invasive capacity (Thakur et al., 2017, Thakur et al., 2021a). These findings suggest that EVs function as mediators linking metabolic stress to tumor progression.

Beyond angiogenesis, EV-mediated protein transfer directly contributes to chemoresistance. GSC-derived EVs contain adenosine-producing enzymes that activate multidrug resistance (MDR) pathways in recipient cells, thereby promoting pharmacological tolerance (Quezada et al., 2018). In parallel, EVs facilitate the horizontal transfer of resistance-related molecules, including ABC transporter family members. For example, transmission of ABCB4 via EVs from glioma stem cells to differentiated glioma cells has been shown to enhance temozolomide resistance through transcriptional regulation mechanisms (Xu X. et al., 2024).

Extracellular vesicles also reinforce canonical DNA repair pathways associated with treatment resistance. GBM-derived exosomes enriched in MGMT and APNG-related transcripts contribute to the repair of alkylating agent-induced DNA damage, although current evidence is largely derived from preclinical models (Shao et al., 2015). This EV-mediated transfer of functional molecules highlights their role as dynamic regulators of therapy response.

Finally, EV-associated proteins such as Notch1 further remodel the tumor microenvironment by promoting angiogenesis, immune suppression, and extracellular matrix reorganization (Sun et al., 2020). Collectively, GBM-derived EV-associated proteins contribute to glioblastoma progression through several interconnected functional axes, including immune evasion, extracellular matrix remodeling, angiogenesis, metabolic adaptation, and therapy resistance. Rather than acting as isolated mediators, these proteins participate in coordinated signaling networks that reshape the tumor microenvironment, enhance tumor plasticity, and promote resistance to chemotherapeutic and immunological stress. Importantly, the translational relevance of these EV-associated proteins varies considerably, with some mechanisms supported primarily by *in vitro* studies, whereas others have been validated in animal models or patient-derived biofluids. A functional overview of major GBM-derived EV-associated proteins, their signaling pathways, mechanistic roles, and current levels of experimental or clinical evidence is summarized in Table 2.

**TABLE 2:** Functional classification of GBM-derived EV-associated proteins involved in immune evasion, chemoresistance, angiogenesis, extracellular matrix remodeling, and glioblastoma progression.

EV-associated protein	Functional category	Recipient cell/signaling pathway	Mechanistic role in GBM progression	Evidence level	EV source	References
PD-L1	Immune checkpoint molecule	T cells/PD-1 signaling	Suppresses T-cell activation and proliferation through PD-1/PD-L1 interaction, contributing to immune evasion and immunosuppressive TME remodeling in GBM	Functional *in vitro* assays, patient-derived EV analysis, and bioinformatic validation	GSC-derived EVs and circulating patient EVs	Ricklefs et al., 2018
Galectin-9 (LGALS9)	Immune checkpoint/ immunosuppressive EV protein	Dendritic cells (TIM-3/LGALS9 axis); downstream suppression of CD8^+^ T-cell activation	GBM-derived exosomal LGALS9 binds TIM-3 on dendritic cells, inhibits antigen uptake/processing/presentation (↓HLA-A, ↓CD40, ↓CD86, ↓TAP1), suppresses cytotoxic T-cell immunity, promotes immune evasion, and tumor progression	Strong experimental evidence (patient CSF proteomics, *in vitro* DC/T-cell assays, CRISPR knockout, mouse GBM models)	GBM-CSF exosomes/GBM cell-derived exosomes	Wang et al., 2020
IDO (IDO1)	Metabolic immunosuppressive enzyme	Tryptophan metabolism/kynurenine pathway affecting T cells	EV-associated IDO promotes tryptophan depletion and kynurenine accumulation, resulting in T-cell dysfunction/exhaustion and establishment of an immunosuppressive GBM microenvironment	Moderate–strong evidence (clinical and mechanistic immunometabolic studies)	GBM-derived extracellular vesicles	Jung et al., 2022
CD39/CD73	Purinergic immune suppression/adenosine pathway	T cells, NK cells, macrophages/adenosine-mediated immunosuppressive signaling	GBM-derived exosomes carry CD39 and CD73 as immunosuppressive protein cargo; these EVs suppress CD8^+^ T-cell and NK-cell activation and promote M2-like macrophage polarization, thereby supporting an immunosuppressive and tumor-promoting microenvironment	*In vitro* + *in vivo*	GB cell line-derived exosomes (U87MG, U251, SBN19)	Azambuja et al., 2020
EGFRvIII	Oncoprotein/invasion-associated EV cargo	GBM recipient cells; EGFR/MAPK, PI3K/Akt, JAK/STAT signaling	EV-mediated transfer of EGFRvIII activates tumor-promoting signaling in recipient GBM cells, enhances invasive phenotype, alters EV biogenesis and uptake, enriches EVs with ECM-binding and pro-invasive proteins (CD44, MCAM, THBS1, ITGA6/ITGB4, PLAU/PLAT), and promotes angiogenesis and tumor dissemination	*In vitro* proteomic + nano-flow cytometry studies	EVs/exosomes derived from EGFRvIII-expressing GBM cells (U373vIII glioma cells)	Choi et al., 2018
PDGFRα-associated signaling	Growth factor receptor signaling	miR-4709-3p/GRB14/PDGFRα pathway	Exosomal circ-METRN activates PDGFRα-associated signaling and promotes glioblastoma progression and radioresistance	Functional validation in GBM models and clinical samples	Glioblastoma-derived exosomes	Wang X. et al., 2021
VEGF	Angiogenesis	Endothelial cells/VEGF–VEGFR2 signaling pathway	GSC-derived exosomes enriched with VEGF and miR-21 enhance endothelial cell migration, tube formation, and angiogenic activity through activation of VEGF/VEGFR2 signaling	*In vitro*	Glioma stem cell-derived exosomes (GSC-EXs) acting on endothelial cells	Sun et al., 2017
HSP27	Stress response/apoptosis resistance	TMZ-induced stress response pathways	TMZ treatment increased HSP27 expression in LN229 glioblastoma cells, suggesting a role in cellular stress adaptation and chemoresistance. However, EV-associated HSP27 could not be detected in EV cargo.	*In vitro* (Western blot analysis)	EVs derived from TMZ-treated U87-MG and LN229 glioblastoma cells	Kıyga et al., 2022
MMP9	Extracellular matrix remodeling	Astrocytes/CD147–JNK signaling pathway	GBM-derived EVs enhanced active MMP9 secretion in astrocytes, promoting extracellular matrix degradation and invasive tumor behavior; the effect was amplified after irradiation	*In vitro* mechanistic study (T98G, U-87 MG, astrocytes)	Glioblastoma-derived extracellular vesicles enriched in CD147 after irradiation	Colangelo and Azzam, 2020
CD133	Stem cell	Stemness-associated signaling	Exosomal CD133 was associated with glioblastoma stem-like characteristics and tumor aggressiveness	*In vivo* (mouse model) + clinical EV detection	GBM-derived extracellular vesicles	Thakur et al., 2021b
CD44	Extracellular matrix	Cell adhesion/migration pathways	Lactate-associated EV signaling increased CD44 expression and EV release, contributing to malignant progression	*In vitro* + *in vivo*	GBM-derived extracellular vesicles	Thakur et al., 2021b
MCT1	Metabolic reprogramming/tumor progression	Neighboring glioma cells and TME metabolic coupling	Hypoxic glioma cells release exosomes enriched with MCT1, supporting glycolytic reprogramming, lactate transport, and malignant progression	*In vitro* molecular and functional studies	Hypoxic glioma-derived exosomes	Thakur et al., 2020
CD147 (EMMPRIN)	Extracellular matrix remodeling/invasion	Astrocytes; JNK/MMP9 signaling	GBM-derived EVs transfer highly glycosylated CD147 to astrocytes, increasing active MMP9 secretion and enhancing invasive extracellular matrix remodeling, especially after irradiation	*In vitro* functional assays	Irradiated and non-irradiated GBM-derived EVs	Colangelo and Azzam, 2020
ABCB4	Chemoresistance/tumor progression	Differentiated glioma cells (DGCs); TMZ resistance pathway	GSC-derived exosomes transfer ABCB4 to DGCs, increasing temozolomide resistance and promoting GBM recurrence; ABCB4 transcription is regulated by ATF3	*In vitro* + *in vivo* xenograft model	Glioblastoma stem cell (GSC)-derived exosomes	Xu X. et al., 2024
RAC1	Immune modulation/macrophage polarization	Microglia; RAC1/AKT/NRF2 signaling pathway	GBM-derived exosomes carrying RAC1 induce microglial M2 polarization through AKT activation and NRF2 nuclear translocation, promoting an immunosuppressive TME	*In vitro* + mouse glioma xenograft model	GBM-derived exosomes	Wu et al., 2025
TREM1	Immune modulation/microglial reprogramming	Microglia; TREM1/SYK-PDK-STAT3 axis	ZIP4-high GBM cells secrete TREM1-enriched EVs that activate ERK1/2 and STAT3 signaling in microglia, inducing IL-6/IL-10 secretion and creating a protumorigenic immune microenvironment	*In vitro* + orthotopic mouse model + patient GBM tissues	GBM cell-derived EVs	Zhang et al., 2025
Notch1	Stemness/tumor progression	Glioma cells/Notch1 signaling pathway	GSC-derived exosomes transfer Notch1 protein to recipient glioma cells, activating the Notch signaling pathway and enhancing stemness, proliferation, invasion, neurosphere formation, and tumorigenicity	*In vitro* + *in vivo* xenograft model	Glioblastoma stem cell (GSC)-derived exosomes	Sun et al., 2020

EVs, extracellular vesicles; GBM, glioblastoma; GSCs, glioma stem-like cells; TME, tumor microenvironment; ECM, extracellular matrix; PD-L1, programmed death-ligand 1; LGALS9, galectin-9; IDO1, indoleamine 2,3-dioxygenase 1; VEGF, vascular endothelial growth factor; PDGFRα, platelet-derived growth factor receptor alpha; EGFRvIII, epidermal growth factor receptor variant III; MMP9, matrix metalloproteinase 9; HSP27, heat shock protein 27; MCT1, monocarboxylate transporter 1; EMT, epithelial–mesenchymal transition; TMZ, temozolomide; AKT, protein kinase B; NRF2, nuclear factor erythroid 2-related factor 2; PI3K, phosphoinositide 3-kinase; JAK/STAT, Janus kinase/signal transducer and activator of transcription; JNK, c-Jun N-terminal kinase; STAT3, signal transducer and activator of transcription 3; ERK1/2, extracellular signal-regulated kinase 1/2; TIM-3, T-cell immunoglobulin and mucin-domain containing-3; NK, natural killer; DC, dendritic cell; HLA-A, human leukocyte antigen A; TAP1, transporter associated with antigen processing 1; CSF, cerebrospinal fluid; DLL1, Delta-like ligand 1; HES1, hairy and enhancer of split-1; HEY1, hes-related family bHLH transcription factor with YRPW motif 1.

Overall, the studies summarized in Table 2 indicate that GBM-derived EV proteins predominantly converge on four major biological programs: (i) suppression of anti-tumor immunity, (ii) enhancement of angiogenesis and metabolic adaptation, (iii) extracellular matrix remodeling and invasion, and (iv) promotion of therapeutic resistance and tumor recurrence.

#### 3.6.3 Lipid composition from glioblastoma-EVs

Glioblastoma-derived extracellular vesicles (EVs) are enriched in specific lipid classes, including saturated fatty acids, sphingomyelin, and sphingosine-1-phosphate (S1P), reflecting the altered sphingolipid metabolism observed in glioblastoma (Haraszti et al., 2016; Sousa et al., 2023).These lipid components have been associated with key tumor-related processes such as proliferation, invasion, and angiogenesis, although most evidence derives from cellular and preclinical models (Sousa et al., 2023).

Recent multi-omics analyses further demonstrate that small EVs (sEVs) derived from glioblastoma stem-like cells are enriched in saturated fatty acids and metabolites involved in lipid and energy metabolism, supporting their role as metabolic carriers between tumor cells (Lokumcu et al., 2024). In particular, metabolites such as glycerol and 2,2-dimethylpropane-1,3-diol identified in GBM-sEVs have been linked to glycerolipid metabolism pathways involved in membrane remodeling and cellular energy balance (Lokumcu et al., 2024)

Given that glioblastoma stem-like cells rely heavily on lipid metabolism for survival under stress conditions, EV-mediated transfer of lipid-associated metabolites may contribute to metabolic adaptation and tumor heterogeneity in recipient cells. However, the extent to which EV lipid cargo directly drives therapy resistance or metabolic reprogramming *in vivo* remains incompletely understood and requires further investigation (Lokumcu et al., 2024).

Importantly, most mechanistic insights into GBM-derived EV cargo—whether protein or lipid—have been obtained from *in vitro* cell lines or patient-derived glioma stem cell models, with only partial validation in animal systems. Clinical studies analyzing EV lipid composition directly from patient biofluids are still limited, highlighting that many proposed EV-mediated mechanisms, including metabolic modulation and therapy resistance, remain at the preclinical stage (Sousa et al., 2023).

## 4 Challenges in GBM treatment and EV-mediated resistance

### 4.1 Challenges in GBM treatment

Despite recent advances in glioblastoma (GBM) therapy, clinical outcomes remain poor due to extensive intratumoral heterogeneity, adaptive resistance mechanisms, and limited drug penetration into the brain parenchyma. Current therapeutic strategies include surgery followed by radiotherapy and systemic treatments such as bevacizumab, erlotinib, immune checkpoint inhibitors, irinotecan, and alkylating agents (Hong et al., 2024). However, among currently available chemotherapeutics, temozolomide (TMZ) remains the standard-of-care agent because of its relatively favorable blood-brain barrier (BBB) permeability and ability to induce DNA damage-mediated programmed cell death (PCD) (Hong et al., 2024; Silva and Colquhoun, 2023; Tomar et al., 2021). TMZ primarily exerts its cytotoxic effects through methylation of DNA bases, particularly at the O6 position of guanine, leading to DNA mismatch formation, replication stress, cell-cycle arrest, and apoptotic signaling (Tomar et al., 2021). Nevertheless, both intrinsic and acquired TMZ resistance substantially limit its therapeutic efficacy. Multiple resistance mechanisms have been implicated, including elevated expression of O6-methylguanine-DNA methyltransferase (MGMT), activation of the base excision repair (BER) pathway, adaptive autophagy, glioma stem cell-associated survival programs, and enhanced drug efflux activity (Hong et al., 2024; Tomar et al., 2021; Yang E. et al., 2022). Among these, MGMT remains one of the most clinically relevant predictive biomarkers, as its overexpression directly reverses TMZ-induced DNA alkylation lesions and reduces treatment responsiveness (Tomar et al., 2021).

Increasing evidence also highlights the contribution of membrane-associated drug transport and extracellular vesicle (EV)-mediated export to TMZ resistance. Drug efflux transporters, including P-glycoprotein and multidrug and toxin extrusion transporter 1 (MATE1/SLC47A1), regulate intracellular drug accumulation and may decrease TMZ retention within GBM cells (Batara et al., 2023; Wang R. et al., 2022). Notably, elevated SLC47A1 expression has been associated with glioma stem cell maintenance, poor prognosis, and reduced TMZ sensitivity, while genetic or pharmacological inhibition of SLC47A1 enhances TMZ responsiveness in preclinical models (Batara et al., 2023).

In parallel, recent evidence suggests that EV secretion may facilitate chemoresistance by promoting extracellular drug export and intercellular transfer of resistance-associated molecules (Yang E. et al., 2022). Mechanistically, disruption of the PTRF/Cavin1-caveolin-1 signaling axis has been shown to reduce small EV secretion, impair TMZ efflux, and enhance intracellular drug accumulation (Yang E. et al., 2022). Similarly, chloroquine-mediated inhibition of autophagy and EV release has demonstrated TMZ-sensitizing effects in experimental GBM models (Yang E. et al., 2022). However, these findings remain largely restricted to *in vitro* and early preclinical investigations, and their translational applicability requires further validation.

Another major obstacle in GBM therapy is the persistence of the blood-brain barrier and the blood-brain tumor barrier (BBTB), both of which continue to restrict effective drug delivery despite tumor-associated vascular abnormalities (Hsu et al., 2024). Although pathological remodeling increases vascular permeability in some GBM regions, heterogeneous BBB disruption and active drug efflux mechanisms still markedly limit therapeutic accumulation within infiltrative tumor areas (Hsu et al., 2024). Consequently, multiple delivery strategies targeting BBB-associated receptors, including transferrin, integrin, and low-density lipoprotein receptors, have been investigated to improve drug penetration and tumor selectivity (Hsu et al., 2024).

In this context, extracellular vesicle-based delivery systems have emerged as promising therapeutic platforms because of their intrinsic biocompatibility, tumor-targeting potential, and ability to cross the BBB. A recent preclinical study demonstrated that umbilical cord mesenchymal stem cell-derived EVs engineered to co-deliver miR-124 and PD-1 induced GBM cell apoptosis, suppressed tumor progression, and enhanced anti-tumor immune responses through modulation of the tumor microenvironment (Yueh et al., 2025). Specifically, this dual-gene delivery strategy promoted T-cell and dendritic cell activation while reducing immunosuppressive cell populations, including regulatory T cells and myeloid-derived suppressor cells (Yueh et al., 2025). Nevertheless, despite encouraging preclinical findings, EV-based therapeutic delivery strategies still face several translational challenges, including large-scale production, cargo heterogeneity, biodistribution control, and long-term safety evaluation before clinical implementation can be achieved.

### 4.2 Alteration of the tumor microenvironment and chemoresistance in GBM

Extracellular vesicles (EVs) are increasingly recognized as critical mediators of glioblastoma (GBM) chemoresistance through their capacity to dynamically remodel the tumor microenvironment (TME). Beyond their role in intercellular communication, EVs regulate immune suppression, angiogenesis, extracellular matrix (ECM) remodeling, and stromal cell reprogramming, thereby promoting tumor progression and therapeutic resistance (Dai et al., 2024; Krapež et al., 2022).

One of the principal mechanisms by which EVs contribute to chemoresistance involves modulation of the immune microenvironment. GBM-derived EVs interact with multiple immune cell populations, including macrophages, monocytes, dendritic cells, and T lymphocytes, resulting in the establishment of an immunosuppressive TME (Dai et al., 2024; Krapež et al., 2022). These interactions impair cytotoxic T-cell activation and proliferation, promote T-cell exhaustion and apoptosis, and facilitate immune evasion through polarization of tumor-associated macrophages (TAMs) toward an M2-like phenotype (Dai et al., 2024; Krapež et al., 2022). Hypoxic conditions within the GBM microenvironment further amplify these effects by altering EV cargo composition, including proteins, cytokines, and non-coding RNAs that enhance TAM polarization and suppress anti-tumor immune responses (Dai et al., 2024; Krapež et al., 2022).

Hypoxia-associated EV signaling also contributes substantially to angiogenesis, which represents a major driver of GBM progression and treatment resistance. Cancer stem cell (CSC)-derived EVs have been shown to promote neovascularization through enrichment of pro-angiogenic mediators, including VEGFA, VEGF-C, EGFR variant III (EGFRvIII), and connexin 43 (Cx43), particularly under hypoxic conditions (Bouzari et al., 2022; Dai et al., 2024; Yang Z. J. et al., 2022). Mechanistically, these EVs modulate endothelial cell gene expression, enhance pericyte-mediated vascular stabilization, and activate oncogenic signaling pathways such as EGFR/MAPK and PI3K/Akt through suppression of ERBB receptor feedback inhibitor 1 (ERRFI1) and phosphatase and tensin homolog (PTEN) (Bouzari et al., 2022; Yang Z. J. et al., 2022). Experimental evidence further demonstrates that hypoxia-derived GBM EVs increase endothelial cell proliferation, migration, and tube formation, supporting their role in pathological angiogenesis and vascular remodeling within the TME (Bouzari et al., 2022; Dai et al., 2024). Notably, exosomal Cx43 has been implicated in facilitating EV uptake by endothelial cells and promoting angiogenic signaling under hypoxic conditions, suggesting its potential relevance as a therapeutic target in GBM-associated neovascularization (Yang Z. J. et al., 2022).

In addition to immune and vascular remodeling, EV-mediated communication between GBM cells and astrocytes contributes to the development of a tumor-supportive microenvironment. Astrocytes exposed to GBM-derived EVs undergo phenotypic and functional alterations that enhance tumor progression, including ECM remodeling, neuroprotective disruption, and promotion of angiogenesis (Dai et al., 2024). Altered EV cargo, including reduced TP53-associated signaling, has been implicated in the conversion of normal astrocytes into reactive tumor-associated astrocytes capable of supporting GBM cell survival and chemoresistance (Dai et al., 2024).

Mesenchymal stem cells (MSCs) and cancer-associated fibroblast (CAF)-like stromal populations also contribute to GBM chemoresistance through complex bidirectional interactions with tumor cells. Although the precise role of MSCs in GBM progression remains controversial, increasing evidence suggests that GBM-derived EVs and soluble factors can induce MSC reprogramming toward CAF-like phenotypes via TGF-β-dependent signaling pathways (Bajetto et al., 2020; Xu J. et al., 2024). These stromal alterations promote extracellular matrix deposition, fibrosis-associated remodeling, maintenance of glioma stemness, and impaired penetration of chemotherapeutic agents within tumor tissues (Bajetto et al., 2020; Xu J. et al., 2024). Furthermore, MSC-derived secretomes and EVs may modulate inflammatory signaling, epithelial-to-mesenchymal transition-like programs, and stemness-associated pathways, thereby further reinforcing treatment resistance and tumor aggressiveness (Bajetto et al., 2020). Nevertheless, the dual tumor-suppressive and tumor-promoting roles of MSCs remain incompletely understood and appear to depend on tumor subtype, MSC origin, and local microenvironmental conditions (Bajetto et al., 2020). Collectively, these findings highlight that EV-mediated remodeling of the GBM microenvironment represents a multifactorial process involving coordinated immune suppression, angiogenesis, stromal reprogramming, and extracellular matrix remodeling. These interconnected mechanisms ultimately facilitate tumor adaptation, therapeutic resistance, and disease progression, emphasizing the importance of targeting EV-associated signaling networks as a potential strategy to overcome GBM chemoresistance.

### 4.3 EVs and immune modulation

Immune evasion is a hallmark of glioblastoma (GBM) progression and represents a major barrier to effective therapy. The process of cancer immunoediting is generally described in three sequential phases: elimination, equilibrium, and escape. During tumor progression, GBM cells progressively acquire mechanisms that suppress anti-tumor immunity and ultimately establish a profoundly immunosuppressive tumor microenvironment (TME), favoring immune escape and therapeutic resistance (Koebel et al., 2007; Takeda et al., 2023). Within this context, extracellular vesicles (EVs) have emerged as key regulators of immune reprogramming through their ability to transfer immunomodulatory proteins, cytokines, lipids, and nucleic acids between tumor cells and immune populations.

The GBM microenvironment contains a heterogeneous population of immune and stromal cells, including T lymphocytes, regulatory T cells (Tregs), natural killer (NK) cells, dendritic cells (DCs), macrophages, microglia, monocytes, myeloid-derived suppressor cells (MDSCs), and granulocytes (Tauriello and Batlle, 2016). Although these immune populations initially contribute to tumor surveillance, GBM-derived EVs actively reshape their phenotypes and functions toward immunosuppressive and tumor-supportive states (Tauriello and Batlle, 2016). EV-mediated signaling therefore represents a critical mechanism through which GBM interferes with both innate and adaptive anti-tumor immune responses (Maacha et al., 2019).

Accumulating evidence indicates that GBM-derived EVs predominantly exert immunosuppressive rather than immunostimulatory effects. These vesicles carry multiple inhibitory molecules, including Fas ligand (FasL), TRAIL, programmed death-ligand 1 (PD-L1), cytotoxic T-lymphocyte-associated protein 4 (CTLA-4), CD39, CD73, transforming growth factor-β (TGF-β), and immunosuppressive non-coding RNAs (Azambuja et al., 2020; Lunavat et al., 2023; Maacha et al., 2019). GBM-derived exosomes (GBex) suppress CD8+ T-cell activation and proliferation, inhibit IFN-γ and TNF-α secretion, induce T-cell apoptosis, and impair NK-cell cytotoxicity (Azambuja et al., 2020). In parallel, GBex promote expansion of Tregs and MDSCs while enhancing M2-like polarization of tumor-associated macrophages (TAMs), thereby reinforcing a highly suppressive immune landscape (Azambuja et al., 2020; Maacha et al., 2019). Mechanistically, several of these effects involve activation of NF-κB signaling pathways in macrophages and modulation of cytokine secretion profiles, including increased IL-10 and TGF-β production together with suppression of pro-inflammatory mediators such as IL-12 and TNF-α (Azambuja et al., 2020; Lunavat et al., 2023).

Immune checkpoint regulation represents another major mechanism of EV-mediated immune suppression in GBM. EV-associated PD-L1 directly interacts with PD-1-expressing T cells, impairing T-cell receptor signaling, clonal expansion, and cytotoxic activity (Lunavat et al., 2023). Similarly, EV-associated CTLA-4 suppresses activation of both CD4+ T cells and NK cells. At the same time, CD39 and CD73 promote extracellular adenosine accumulation, further inhibiting T-cell proliferation and effector function through adenosine receptor signaling pathways (Lunavat et al., 2023). GBM-derived EVs also transport immunosuppressive microRNAs, including miR-21, miR-29a, miR-92a, miR-1246, and miR-10a, which collectively contribute to the establishment of an “immunosuppressive halo” surrounding tumor cells (Lunavat et al., 2023).

Beyond direct immune suppression, GBM-derived EVs alter antigen presentation and immune cell differentiation within the TME. EV-mediated transfer of galectin-9 suppresses dendritic cell antigen processing and presentation through interaction with TIM-3 signaling pathways, thereby impairing downstream cytotoxic T-cell responses (Lunavat et al., 2023). Additionally, GB-derived EVs can induce differentiation of monocytes toward immunosuppressive myeloid-derived suppressor cell phenotypes and interfere with normal dendritic cell maturation (Lunavat et al., 2023; Maacha et al., 2019). Recent findings further suggest that interferon-γ-induced release of PD-L1- and indoleamine 2,3-dioxygenase (IDO)-containing EVs contributes to “superinduction” mechanisms that amplify immune suppression through reciprocal communication between tumor cells and myeloid populations (Lunavat et al., 2023).

Importantly, EV-mediated immune modulation in GBM is highly context dependent and influenced by the cellular origin of EVs, hypoxic conditions, treatment exposure, and dynamic interactions within the TME (Lunavat et al., 2023). While most studies support a predominantly tumor-promoting role for GBM-derived EVs, emerging evidence suggests that certain EV populations released by immune or stromal cells may exert anti-tumor functions under specific conditions (Lunavat et al., 2023). Nevertheless, the overall balance within the GBM microenvironment strongly favors immune suppression, facilitating tumor progression, resistance to immunotherapy, and disease recurrence.

Collectively, these findings highlight EVs as central orchestrators of immune dysregulation in GBM. By simultaneously targeting T cells, NK cells, macrophages, dendritic cells, and myeloid suppressor populations, GBM-derived EVs establish a coordinated immunosuppressive network that promotes immune escape and limits therapeutic efficacy. Accordingly, targeting EV biogenesis, cargo loading, or EV-mediated immune checkpoint signaling has emerged as a promising strategy to improve immunotherapeutic responses in GBM.

#### 4.3.1 Macrophages and microglia

A major immunomodulatory effect of GBM-EVs involves the reprogramming of macrophages and microglia toward tumor-supportive and immunosuppressive phenotypes. Experimental evidence indicates that GBM-derived exosomes carrying RAC1 promote microglial M2-like polarization through activation of the RAC1/AKT/NRF2 signaling pathway (Wu et al., 2025). Similarly, ZIP4 has been identified as a regulator of EV-mediated tumor–microglia communication in GBM. ZIP4-high GBM cells release TREM1-enriched EVs through ZEB1-dependent regulation, and EV-derived TREM1 activates SYK/PDK/STAT3 signaling in microglia, promoting an immunosuppressive microenvironment. Inhibition of ZIP4 or TREM1 attenuated tumor growth in orthotopic mouse models, supporting the functional relevance of this pathway (Zhang et al., 2025).

Glioblastoma -EVs are efficiently internalized by monocytes, macrophages, and microglia, leading to phenotypic and functional reprogramming of myeloid cells (Azambuja et al., 2020; Krapež et al., 2022). Exposure to GBM-EVs promotes the acquisition of immunosuppressive macrophage features, including increased expression of CD163, CD206, IL-10, VEGF, PD-1, CD39, and PD-L1-associated suppressive phenotypes (Azambuja et al., 2020; Krapež et al., 2022). In parallel, PD-L1 carried by GBM-EVs can interact with PD-1 on activated T cells and contribute to impaired T-cell activation and proliferation (Krapež et al., 2022). Ionizing radiation further enhances EV-mediated immunosuppression by increasing PD-L1 expression and enriching CD206-positive macrophages, thereby promoting a pro-oncogenic microenvironment (Schweiger et al., 2024). However, most of these findings are derived from preclinical glioma models and require validation across molecularly diverse GBM subtypes and patient-derived systems.

Glioblastoma-derived exosomes also activate NF-κB signaling in macrophages, contributing to M2-like polarization; inhibition of NF-κB reverses this phenotype (Azambuja et al., 2020). *In vivo* administration of GBM-derived exosomes reduced splenic CD8+ T cells, NK cells, and M1-like macrophages while increasing naïve and M2-like macrophage populations (Azambuja et al., 2020).

Therapeutically, engineered EV-based approaches may partially reverse macrophage-mediated immunosuppression. A recent preclinical study demonstrated that folate-modified EVs delivering miR-138 reduced CD206+ PD-L1+ M2 macrophages and TGF-β levels while increasing IL-1β expression and CD8+ T-cell infiltration, thereby shifting the GBM microenvironment toward a more pro-inflammatory state (Nguyen et al., 2025).

Hypoxic GBM-derived EVs enriched in miR-1246 further promote M2-like polarization in tumor-associated macrophages through modulation of STAT3 and NF-κB signaling pathways (Qian et al., 2020). In addition, EV-mediated transfer of miR-451 and miR-21 from GBM cells to microglia suppresses c-Myc expression, contributing to immune dysregulation within the tumor microenvironment (Musatova and Rubtsov, 2023). GBM-EVs may also impair antigen presentation by reducing HLA-DR expression and co-stimulatory molecules in macrophages and monocytes (Iorgulescu et al., 2016; Krapež et al., 2022).

#### 4.3.2 MDSCs

Glioblastoma-EVs significantly contribute to MDSC expansion and activation within the TME. MDSCs represent a major immunosuppressive myeloid population within the GBM microenvironment and suppress anti-tumor immune responses through ARG1 activity, immune checkpoint signaling, and secretion of immunosuppressive cytokines (Sharma et al., 2023). MDSCs suppress immune responses through IL-10 and TGF-β, and their induction is mediated by PD-L1 and IDO1 present in GBM-EVs (Jung et al., 2022). MDSCs internalize EVs at rates comparable to macrophages and microglia and can also release PD-L1–loaded EVs (Ricklefs et al., 2018). Under hypoxic conditions, GBM releases increased quantities of EVs with distinct mRNA profiles that promote MDSC production, resulting in arginine depletion and inhibition of T-cell activation (Grzywa et al., 2020). Hypoxic GBM-EVs also transfer miR-10a and miR-21, which suppress PTEN and RORα signaling pathways, activating MDSCs via the PTEN/PI3K/AKT and RORα/IκBα/NF-κB axes (Guo et al., 2018).

#### 4.3.3 Dendritic cells

In GBM patients, dendritic cells are increased in cerebrospinal fluid; however, GBM-EVs impair their antigen-presenting capacity. LGALS9 present in GBM-EVs inhibits antigen processing and presentation, and interaction with Siglec-9 further suppresses DC immune function (Wang X. et al., 2021). Defective dendritic-cell maturation and impaired antigen presentation are important contributors to the profoundly immunosuppressive GBM microenvironment and result in insufficient T-cell priming and weak anti-tumor immunity (Sharma et al., 2023).

#### 4.3.4 Natural killer cells

The glioma TME reduces NK-cell functionality primarily through TGF-β secretion, decreasing NKG2D receptor expression in NK cells from GBM patients (Castriconi et al., 2009; Sharma et al., 2023). In addition to functional suppression, NK cells are relatively scarce within the GBM microenvironment, further limiting effective innate anti-tumor immunity (Sharma et al., 2023). GBM-EVs reduce splenic CD8^+^ T-cell numbers and diminish granzyme B and IFN-γ secretion (de Vrij et al., 2015). FasL expressed by GBM may induce T-cell apoptosis via the Fas/FasL pathway *in vitro* and *in vivo* (Abusamra et al., 2005; Liu et al., 2013).

#### 4.3.5 T cells and Tregs

Vesicular LGALS9 (Galectin-9) binds TIM-3 on CD4^+^ T cells, inducing apoptosis and contributing to CD8^+^ T-cell exhaustion (Liang et al., 2019; Wang et al., 2020). GBM-EVs also contain TGF-β, further inhibiting T-cell function (Graner et al., 2009). GBM-EVs expressing CD73 and CD39 enhance adenosine production following uptake by T cells and Tregs, thereby suppressing glycolysis, clonal expansion, and effector T-cell metabolism (Wang M. et al., 2021). In addition, PD-L1/PD-1–containing GBM-derived exosomes promote Treg-mediated immunosuppression and inhibit effector T-cell activity through immune checkpoint signaling (Li P. et al., 2017).

#### 4.3.6 B cells

Glioblastoma-derived EVs containing placental growth factor (PlGF) stimulate naïve B-cell differentiation into regulatory B cells (Bregs), which suppress CD8^+^ T-cell proliferation and cytotoxic function, thereby further reinforcing the immunosuppressive tumor microenvironment (Han et al., 2014).

## 5 Therapeutic targeting of EVs in glioblastoma

### 5.1 Inhibition of EV release

Tumor-derived EVs facilitate the local and systemic transfer of bioactive molecules, thereby modulating the tumor microenvironment (TME). In addition, tumor-derived EVs (TDEs) promote the proliferation and metastasis of recipient tumor cells (Irep et al., 2024). Glioma-derived EVs (GDEs) have been associated with angiogenesis, enhanced migration, increased glioma cell proliferation, tumor invasiveness, and activation of multiple oncogenic signaling pathways. Furthermore, GDEs have been implicated in glioma initiation, progression, and therapeutic resistance (Shi et al., 2020). As key components of the TME, EVs contribute to multiple stages of cancer progression, including angiogenesis, metastasis, immune modulation, and therapeutic resistance (Zou et al., 2025a).

In cancer and other pathological conditions, targeting small extracellular vesicle (sEV) biogenesis and secretion has emerged as a potential strategy to reduce tumor progression, overcome therapeutic resistance, and improve treatment efficacy. Inhibition of sEV secretion may alter intercellular communication within the tumor microenvironment and has shown anti-tumor effects in several preclinical models. Inhibition of neutral sphingomyelinase 2 (nSMase2), a key enzyme involved in the ceramide-dependent pathway of sEV biogenesis and release, has been experimentally achieved using compounds such as GW4869 and manumycin A (Shams et al., 2024). Additionally, several small-molecule inhibitors (SMIs) have been reported, including the nSMase2 inhibitor DPTIP 2,6-Dimethoxy-4-(5-Phenyl-4-(Thiophen-2-yl)-1H-Imidazole-2-yl)Phenol, glibenclamide (an anti-diabetic drug), imipramine (an antidepressant), simvastatin (an HMG-CoA reductase inhibitor), dimethyl amiloride, ketoconazole (an antifungal), omeprazole (a proton-pump inhibitor), and cannabidiol, all of which have been reported to modulate or inhibit EV biogenesis and/or release in different experimental models (Table 3; Shams et al., 2024). Biotechnological approaches such as RNA interference (RNAi) and the CRISPR-Cas9 system can be employed to modulate or inhibit the expression of genes involved in TDE biogenesis and secretion (Wang M. et al., 2021). For example, RNAi-mediated silencing of 23 components of the ESCRT machinery in HeLa-CIITA cells has been shown to reduce EV secretion (Li J. et al., 2022).

**TABLE 3:** Representative extracellular vesicle-associated biomarkers implicated in glioblastoma chemoresistance and therapeutic resistance mechanisms.

EV cargo category	Biomarker	Regulation in resistant GBM	Associated chemoresistance mechanism	Potential clinical significance	References
Protein-associated EV biomarkers					
Protein-associated EV biomarkers	ABCB4	Upregulated in GSCs and GSC-derived exosomes from TMZ-resistant GBM models (*in vitro*/*in vivo*)	Exosomal transfer of ABCB4 promotes TMZ resistance through enhanced drug efflux and reduced apoptosis	Candidate biomarker and potential preclinical therapeutic target for TMZ resistance	Xu X. et al., 2024
Protein-associated EV biomarkers	EGFRvIII	Enriched in EVs derived from EGFRvIII-expressing GBM cells (*in vitro*)	Alters EV proteomic composition and promotes pro-invasive signaling associated with aggressive GBM phenotypes	Candidate EV-associated biomarker for aggressive GBM subtypes	Choi et al., 2018
Protein-associated EV biomarkers	CD44	Enriched in EVs derived from EGFRvIII-positive GBM cells (*in vitro*)	Associated with pro-invasive EV phenotypes linked to aggressive tumor behavior	Candidate marker of aggressive GBM-associated EV subpopulations	Oushy et al., 2018
Protein-associated EV biomarkers	HSP70	Highly elevated in plasma-derived EVs from GBM patients before surgery (clinical)	–	Proposed circulating biomarker for GBM characterization and disease monitoring	Alberti et al., 2024
Protein-associated EV biomarkers	HSP90	Detected in GBM-derived EVs/exosomes under hypoxic conditions (preclinical)	Associated with hypoxia-related signaling and stress adaptation	Candidate EV-associated biomarker in hypoxic GBM models	Kucharzewska et al., 2013
Protein-associated EV biomarkers	PD-L1	Expressed on the surface of subsets of GBM-derived EVs and patient-derived EVs (*in vitro*/clinical)	Suppresses T-cell activation and proliferation through PD1-mediated immune evasion	Circulating EV-associated PD-L1 DNA correlated with GBM tumor volume	Ricklefs et al., 2018
miRNA-associated EV biomarkers					
miRNA-associated EV biomarkers	miR-221	Upregulated in TMZ-resistant glioma cells and derived exosomes (*in vitro*)	Exosomal miR-221 promotes glioma cell proliferation, migration, anti-apoptotic activity, and TMZ resistance through DNM3 targeting	Candidate biomarker associated with glioma progression and TMZ resistance	Yang et al., 2017
miRNA-associated EV biomarkers	miR-1238	Upregulated in TMZ-resistant GBM cells and derived exosomes (*in vitro*)	Exosomal miR-1238 transfer promotes TMZ resistance through CAV1/EGFR signaling	Candidate circulating/exosomal biomarker and potential preclinical therapeutic target for TMZ resistance in GBM	Yin et al., 2019
miRNA-associated EV biomarkers	miR-151a	Downregulated in TMZ-resistant GBM cells, recurrent GBM tissues, and CSF-derived exosomes from GBM patients (*in vitro*/*in vivo*/clinical)	Loss of exosomal miR-151a promotes TMZ resistance through XRCC4-mediated DNA repair, whereas restoration enhances TMZ sensitivity	CSF-derived exosomal miR-151a may predict chemotherapy response and represents a candidate therapeutic target for therapy-refractory GBM	Zeng et al., 2018b
miRNA-associated EV biomarkers	miR-25-3p	Upregulated in exosomes from TMZ-resistant GBM cells and serum samples from TMZ-treated GBM patients (*in vitro*/clinical)	Exosomal miR-25-3p promotes TMZ resistance through FBXW7 suppression and activation of c-Myc/cyclin E signaling	Candidate prognostic biomarker associated with TMZ resistance in GBM	Wang J. et al., 2021
miRNA-associated EV biomarkers	miR-30b-3p	Upregulated in EVs derived from hypoxic glioma stem-like cells and detected in CSF samples from GBM patients (*in vitro*/*in vivo*/clinical)	EV-delivered miR-30b-3p promotes TMZ resistance through RHOB suppression, reduced apoptosis, and enhanced proliferation under hypoxic conditions	Potential biomarker for hypoxia-associated TMZ resistance in GBM	Yin et al., 2021
miRNA-associated EV biomarkers	miR-106a-5p	Upregulated in hypoxic glioma cells and hypoxia-derived exosomes (*in vitro*/*in vivo*)	Exosomal miR-106a-5p reduces TMZ sensitivity through PTEN downregulation and Akt pathway activation	Candidate preclinical biomarker and therapeutic target for hypoxia-associated TMZ resistance in GBM	Wu et al., 2022
lncRNA-associated EV biomarkers					
lncRNA-associated EV biomarkers	SBF2-AS1	Upregulated in TMZ-resistant GBM cells, recurrent GBM tissues, and serum-derived exosomes from recurrent GBM patients (*in vitro*/*in vivo*/clinical)	Exosomal SBF2-AS1 functions as a ceRNA for miR-151a-3p, resulting in XRCC4 upregulation, enhanced DNA double-strand break repair, and increased TMZ resistance	Candidate circulating biomarker associated with poor TMZ response in GBM; exosomal SBF2-AS1 may represent a potential preclinical therapeutic target for TMZ-resistant GBM	Zhang et al., 2019
lncRNA-associated EV biomarkers	lnc-TALC	Upregulated in TMZ-resistant GBM cells and enriched in GBM-derived exosomes; transferred to microglia and induces M2 polarization (*in vitro*/*in vivo*)	Exosomal lnc-TALC binds ENO1 and activates the p38 MAPK/MEF2C axis, increasing microglial C5/C5a secretion, which enhances DNA damage repair (ATM/ATR/RAD51) and suppresses TMZ-induced apoptosis, leading to TMZ resistance	Candidate preclinical therapeutic target for TMZ resistance; targeting C5a/C5aR restored TMZ sensitivity *in vitro* and prolonged survival in mouse GBM models	Li et al., 2021
lncRNA-associated EV biomarkers	Lnc-DLK1-35	Enriched in TMZ-resistant GBM cells and derived exosomes (*in vitro*)	Exosomal Lnc-DLK1-35 reprograms microglia toward a pro-tumorigenic phenotype, thereby enhancing GBM invasion and TMZ resistance	Candidate preclinical therapeutic target for modulating GBM–microglia communication and chemoresistance	Li et al., 2026
lncRNA-associated EV biomarkers	HOTAIR	Highly upregulated in GBM tissues, TMZ-resistant GBM cell lines, and serum-derived EVs from GBM patients (*in vitro*/*in vivo*/clinical)	EV-mediated transfer of HOTAIR promotes TMZ resistance through miR-526b-3p sponging and EVA1 upregulation, enhancing GBM proliferation, invasion, and anti-apoptotic signaling	Candidate circulating diagnostic and prognostic biomarker for TMZ-resistant GBM; the HOTAIR/miR-526b-3p/EVA1 axis may represent a potential preclinical therapeutic target	Wang R. et al., 2022
circRNA-associated EV biomarkers					
circRNA-associated EV biomarkers	circWDR62	Upregulated in TMZ-resistant glioma cells, serum exosomes, and recurrent glioma patient tissues (*in vitro*/clinical)	Transfers TMZ resistance via the circWDR62/miR-370-3p/MGMT axis; promotes proliferation, invasion, migration, and malignant progression	Candidate circulating prognostic biomarker and potential preclinical therapeutic target for TMZ-resistant GBM	Geng et al., 2022
circRNA-associated EV biomarkers	circCABIN1	Upregulated in TMZ-resistant GBM cells and derived exosomes (*in vitro*)	Sponges miR-637 to upregulate OLFML3 and activate ErbB signaling, sustaining GSC stemness and TMZ resistance	Candidate predictor of recurrence and potential therapeutic target via engineered exosome-mediated siRNA delivery	Liu et al., 2023
circRNA-associated EV biomarkers	circGLIS3	Upregulated in TMZ-resistant glioma cells and derived exosomes; validated in xenograft models (*in vitro*/*in vivo*)	Regulates the miR-548m/MED31 axis to promote proliferation, invasion, migration, suppress apoptosis, and enhance TMZ resistance	Candidate preclinical biomarker associated with TMZ-resistant glioma	Li and Lan, 2023

ABCB4, ATP-binding cassette subfamily B member 4; ATM, ataxia telangiectasia mutated; ATR, ATM and Rad3-related; BBB, blood–brain barrier; CAV1, caveolin-1; CDK, cyclin-dependent kinase; circRNA, circular RNA; CSF, cerebrospinal fluid; EGFRvIII, epidermal growth factor receptor variant III; ErbB, erythroblastic leukemia viral oncogene homolog; EV, extracellular vesicle; GBM, glioblastoma; GSC, glioma stem cell; HSP70, heat shock protein 70; HSP90, heat shock protein 90; lncRNA, long non-coding RNA; MAPK, mitogen-activated protein kinase; MEF2C, myocyte enhancer factor 2C; MGMT, O6-methylguanine-DNA methyltransferase; miRNA, microRNA; OS, overall survival; PD-L1, programmed death-ligand 1; PTEN, phosphatase and tensin homolog; RAD51, RAD51 recombinase; siRNA, small interfering RNA; TMZ, temozolomide; XRCC4, X-ray repair cross-complementing protein 4.

In the process leading to TDE release, secretory multivesicular bodies (MVBs) are transported to the cell periphery and dock with the plasma membrane, a process regulated by members of the Rab GTPase family, particularly Rab27a and Rab27b, which regulate multivesicular body trafficking, docking, and fusion with the plasma membrane during EV secretion. Rab27a primarily governs the docking and fusion of MVBs with the plasma membrane (Blanc and Vidal, 2018). Hypoxia and extracellular acidosis are key microenvironmental factors that regulate the biogenesis and secretion of tumor-derived EVs within the TME (Xi et al., 2021).

Under hypoxic conditions, hypoxia-inducible factor-1α (HIF-1α) promotes pyruvate kinase M2 (PKM2)-mediated phosphorylation of synaptosome-associated protein 23 (SNAP-23) at Ser95, thereby facilitating TDE secretion through SNARE complex activation. In acidic microenvironments, caveolin-1-mediated regulation of membrane cholesterol dynamics has also been implicated in enhanced EV formation and TDE secretion (Xi et al., 2021). For example, downregulation of ATP9A expression in cancer cells has been associated with increased sEV secretion (Naik et al., 2019).

While targeting extracellular vesicle (EV) release represents a promising therapeutic strategy, several critical limitations must be considered. EV secretion is a fundamental and ubiquitous biological process occurring in both physiological and pathological conditions, with vesicles being released by a wide variety of normal cell types and participating in essential intercellular communication pathways (Kumar et al., 2024). Therefore, non-selective inhibition of EV biogenesis or release may disrupt normal cellular homeostasis and interfere with vital signaling mechanisms.

In addition, EV populations are highly heterogeneous in terms of size, cellular origin, molecular composition, and functional properties, which complicates their uniform targeting and challenges the assumption that EVs uniformly exert tumor-promoting effects (Primorac et al., 2026; Zou et al., 2025b). This heterogeneity is further influenced by the physiological state of the parental cells and environmental conditions, resulting in substantial variability in EV cargo and biological activity (Kumar et al., 2024; Primorac et al., 2026).

Moreover, current strategies for inhibiting EV production or engineering EV-based therapeutics face significant technical and translational limitations, including low targeting efficiency, rapid clearance, non-specific uptake by organs such as the liver and spleen, and variability in cargo loading and delivery efficiency (Ma et al., 2025). Bioengineering approaches intended to enhance EV functionality may also introduce additional concerns such as byproduct contamination, instability, and potential off-target biological effects (Ma et al., 2025).

Finally, despite increasing interest and promising preclinical findings, the clinical translation of EV-targeting strategies remains limited. Most EV-based therapeutic approaches are still in early-stage clinical development, with major unresolved challenges related to standardization, regulatory approval, safety evaluation, and large-scale production (Zou et al., 2025b). Collectively, these limitations underscore the importance of developing selective and context-specific strategies for targeting EVs, rather than relying on indiscriminate suppression of EV biogenesis and release in glioblastoma.

### 5.2 EV-based biomarkers

Given the highly aggressive and heterogeneous nature of glioblastoma (GBM), early diagnosis, longitudinal disease monitoring, and accurate assessment of therapeutic response remain major clinical challenges. Conventional diagnostic approaches, including tissue biopsy and magnetic resonance imaging (MRI), are associated with several limitations. Tissue biopsies are invasive and provide only a static representation of a highly dynamic tumor, whereas imaging modalities often fail to reliably distinguish true tumor progression from treatment-related pseudoprogression (Cela et al., 2024; Zanganeh et al., 2023). These limitations have accelerated interest in liquid biopsy approaches as minimally invasive strategies for real-time monitoring of tumor evolution and therapeutic response.

Among currently investigated liquid biopsy analytes, circulating tumor cells (CTCs), circulating tumor DNA (ctDNA), cell-free RNAs (cfRNAs), and extracellular vesicles (EVs) have emerged as promising candidates for GBM detection and monitoring in body fluids such as blood and cerebrospinal fluid (CSF) (Cela et al., 2024; Seyhan, 2024; Zanganeh et al., 2023). These circulating biomarkers provide valuable insights into tumor burden, molecular alterations, disease progression, and therapeutic response in GBM patients (Zanganeh et al., 2023). These include circulating tumor cells (CTCs), circulating cell-free DNA (cfDNA) and RNA (ctRNA), and EVs, which play a key role in tumor biology (Zanganeh et al., 2023).

Extracellular vesicles are considered particularly attractive because they are actively released by tumor cells into biofluids such as blood and cerebrospinal fluid (CSF), while protecting their molecular cargo from enzymatic degradation through their lipid bilayer membrane (Seyhan, 2024). In addition, EVs can cross the blood-brain barrier (BBB), allowing access to tumor-associated molecular information through minimally invasive sampling procedures (Seyhan, 2024; Skouras et al., 2023).

Importantly, the molecular composition of GBM-derived EVs partially reflects the phenotypic and genotypic characteristics of their parental tumor cells. These EVs may contain oncogenic proteins, DNA fragments, mRNAs, microRNAs (miRNAs), long non-coding RNAs (lncRNAs), circular RNAs (circRNAs), lipids, and metabolites (Skouras et al., 2023; Zanganeh et al., 2023). Consequently, EV cargo profiling has been investigated as a potential strategy for molecular subclassification, assessment of tumor burden, prediction of recurrence, and monitoring of therapeutic response in GBM patients (Cela et al., 2024; Seyhan, 2024; Skouras et al., 2023). Several studies have demonstrated that EV concentration (sometimes referred to as “vesiclemia”), EV size distribution, and EV-associated ncRNA signatures may correlate with disease progression, recurrence, and response to chemo-radiotherapy (Cela et al., 2024; Skouras et al., 2023). Notably, increased EV release has been reported in both newly diagnosed and recurrent GBM compared with healthy controls or post-treatment patients, suggesting a potential association between EV dynamics and tumor activity (Skouras et al., 2023; Zhao et al., 2019).

Beyond their diagnostic value, EVs have also been implicated in the development of therapeutic resistance. Experimental studies, primarily derived from *in vitro* and preclinical GBM models, indicate that EV-associated proteins and non-coding RNAs contribute to temozolomide (TMZ) resistance through modulation of DNA repair pathways, apoptosis inhibition, stemness maintenance, metabolic adaptation, and immune reprogramming (Lunavat et al., 2023; Zhao et al., 2019; Zhou et al., 2021). For example, EV-mediated transfer of resistance-associated miRNAs, lncRNAs, circRNAs, and proteins has been shown to enhance glioma cell survival and modulate sensitivity to temozolomide (TMZ) and radiotherapy through mechanisms involving apoptosis inhibition, enhanced DNA repair, stemness maintenance, immune modulation, and metabolic adaptation (Table 3). Accordingly, EV cargo signatures are increasingly being explored as candidate biomarkers for predicting therapeutic response, disease progression, and chemoresistance in GBM patients.

Despite these promising findings, several important translational limitations currently restrict the routine clinical implementation of EV-based biomarkers. First, EV populations exhibit substantial biological heterogeneity, reflecting contributions from both tumoral and non-tumoral cells within the circulation. This significantly complicates the identification of tumor-specific EV signatures, particularly in peripheral blood samples (Seyhan, 2024; Skouras et al., 2023). Second, the lack of standardized protocols for EV isolation, purification, storage, and characterization introduces considerable inter-study variability and limits reproducibility. Moreover, co-isolation of lipoproteins, protein aggregates, and non-vesicular particles may interfere with downstream molecular analyses and reduce diagnostic specificity (Skouras et al., 2023; Zanganeh et al., 2023; Zhao et al., 2019). Additional challenges include low EV yield, short circulating half-life, variability in pre-analytical handling, and the absence of clinically validated large-scale prospective studies.

Therefore, although EVs represent highly promising biomarkers for liquid biopsy-based GBM management, most current evidence remains at the preclinical or exploratory clinical stage. Further multicenter studies, methodological standardization, and prospective clinical validation are required before EV-based biomarkers can be reliably integrated into routine neuro-oncological practice.

Collectively, current evidence indicates that EV-associated biomolecules contribute to GBM chemoresistance through several convergent functional mechanisms, including enhancement of DNA damage repair, inhibition of apoptosis, maintenance of glioma stemness, metabolic adaptation, immune evasion, and activation of pro-survival signaling pathways. Among these EV cargos, proteins and non-coding RNAs—including miRNAs, lncRNAs, and circRNAs— represent some of the most extensively investigated classes of resistance-associated biomarkers in preclinical GBM models and patient-derived biofluids. A summary of representative EV-associated biomarkers implicated in GBM chemoresistance is presented in Table 3.

### 5.3 Translational challenges and clinical-scale barriers

Despite increasing preclinical evidence supporting the therapeutic and diagnostic potential of extracellular vesicles (EVs), multiple translational and clinical-scale barriers continue to limit their routine clinical implementation. These challenges extend beyond proof-of-concept efficacy and primarily involve limitations in large-scale manufacturing, vesicle heterogeneity, cargo reproducibility, biodistribution, and regulatory standardization.

One of the major translational obstacles involves the absence of robust and standardized Good Manufacturing Practice (GMP)-compliant production systems for clinical-grade EVs. Although recent advances in bioreactor technologies and scalable production platforms have improved EV yield and manufacturing feasibility, maintaining batch-to-batch consistency remains difficult because of the intrinsic biological variability of parental cells and the sensitivity of EV secretion to culture conditions, environmental stress, and donor-cell characteristics (Ma et al., 2025; Zou et al., 2025b). In addition, bioengineering strategies designed to enhance EV therapeutic efficacy may further increase manufacturing complexity by altering vesicle composition, introducing unintended byproducts, and generating variability in cargo loading efficiency, thereby complicating quality control and reproducibility (Ma et al., 2025).

Extracellular vesicle heterogeneity represents another major challenge limiting translational reproducibility. Even when derived from a single parental cell population, EVs exhibit considerable diversity in size distribution, membrane composition, surface markers, and molecular cargo content (Ma et al., 2025; Park and Jung, 2025). Such heterogeneity may directly influence therapeutic potency, biodistribution, immune interactions, and targeting specificity. However, the magnitude of these effects can vary depending on EV source, isolation methodology, and recipient tissue context. Furthermore, widely used isolation approaches—including ultracentrifugation, precipitation-based methods, and size-exclusion chromatography—often generate vesicle populations with variable purity profiles and contaminant levels, thereby contributing to inter-study variability and limiting cross-platform standardization (Park and Jung, 2025; Zou et al., 2025b). In addition, differences in pre-analytical handling, storage conditions, and EV characterization protocols further complicate reproducibility and clinical comparability across studies.

Efficient and reproducible cargo loading also remains an unresolved bottleneck for EV-based therapeutic development. Physical loading strategies such as electroporation, sonication, and membrane permeabilization techniques may compromise vesicle membrane integrity and promote nucleic acid aggregation or degradation, raising concerns regarding functional consistency, loading reproducibility, and long-term safety (Ma et al., 2025). Although artificial or hybrid exosome systems have been proposed to improve scalability, engineering flexibility, and cargo uniformity, their biological equivalence, pharmacokinetic behavior, and long-term biosafety require further validation, particularly in clinically relevant *in vivo* systems, before successful clinical translation can be achieved (Park and Jung, 2025).

Another major translational concern involves *in vivo* biodistribution and off-target accumulation. Native EVs are frequently sequestered by the mononuclear phagocyte system, particularly within the liver and spleen, thereby limiting efficient delivery to intended target tissues (Ma et al., 2025). In addition, rapid systemic clearance and non-specific uptake remain major barriers to achieving therapeutically relevant accumulation within intracranial tumors such as glioblastoma. Although surface modification and genetic engineering approaches have been developed to enhance tissue tropism and targeting specificity, these strategies may also introduce additional safety variables, immunogenicity concerns, and toxicological uncertainties that require systematic evaluation before clinical application.

From a regulatory perspective, EV-based therapeutics remain positioned within an evolving and incompletely harmonized framework. Regulatory agencies such as the U.S. Food and Drug Administration (FDA) and the European Medicines Agency (EMA) require clearly defined characterization standards, validated potency assays, and stringent release criteria for clinical-grade EV products (Primorac et al., 2026). However, universally accepted quality-control benchmarks, standardized reference materials, and consensus functional assays for EV products are still lacking (Primorac et al., 2026). In parallel, the Minimal Information for Studies of Extracellular Vesicles (MISEV) recommendations have emphasized the need for standardized EV characterization and reporting practices to improve reproducibility and cross-study comparability. Nevertheless, substantial variability in isolation methodologies, characterization protocols, and manufacturing workflows continues to hinder regulatory harmonization and large-scale commercialization.

Collectively, these challenges highlight that successful clinical translation of EV-based therapeutics will require not only technological optimization, but also harmonized regulatory frameworks, standardized manufacturing pipelines, reproducible functional characterization assays, and rigorous long-term safety evaluation. Addressing these limitations will be essential to bridge the gap between experimental promise and reproducible, safe, and clinically scalable implementation of EV-based therapeutics.

## 6 Conclusion

Glioblastoma remains one of the most therapeutically challenging malignancies because of its remarkable cellular heterogeneity, adaptive plasticity, and highly immunosuppressive tumor microenvironment. In recent years, extracellular vesicles (EVs) have emerged as important mediators of intercellular communication that influence multiple dimensions of GBM biology, including tumor progression, immune modulation, metabolic adaptation, stemness maintenance, and resistance to chemotherapy. Accumulating evidence suggests that EV-associated cargos, including non-coding RNAs, proteins, lipids, and signaling mediators, participate in coordinated resistance programs rather than isolated molecular events and thereby contribute to the dynamic evolution of therapy-resistant phenotypes under treatment pressure.

At the same time, the biological roles of GBM-derived EVs should not be considered universally tumor-promoting or mechanistically uniform. Several findings indicate that EV-mediated effects may vary according to molecular subtype, microenvironmental context, recipient cell population, and experimental setting. Moreover, many proposed EV-associated mechanisms remain predominantly supported by *in vitro* or preclinical evidence, whereas robust clinical validation is still limited. Recognition of these contextual and methodological limitations is essential for accurate interpretation of the translational significance of EV biology in GBM.

Beyond their mechanistic relevance, EVs continue to attract considerable interest as potential biomarkers and therapeutic tools because of their relative stability, capacity to cross biological barriers, and ability to reflect tumor-specific molecular states. Nevertheless, important translational and clinical challenges remain unresolved. These include EV heterogeneity, variability among isolation and characterization methods, limited scalability and reproducibility of production platforms, cargo-loading inefficiency, incomplete regulatory standardization, and the risk of unintended interference with physiological EV-mediated communication. Consequently, indiscriminate inhibition of EV release may not represent a clinically viable strategy, emphasizing the need for selective, context-specific, and biologically informed targeting approaches.

Overall, continued progress in the EV field will require stronger mechanistic integration, standardized methodological frameworks, and more robust clinical validation studies capable of distinguishing biologically meaningful EV signatures from experimental variability. A more refined understanding of how EV-mediated signaling evolves across GBM subtypes and treatment states may ultimately facilitate the development of more precise diagnostic, prognostic, and therapeutic strategies for glioblastoma.

## Glossary

ABCB4: ATP Binding Cassette Subfamily B Member 4
ADAMTS9-AS2,: ADAMTS9 Antisense RNA 2;
AFM,: Atomic Force Microscopy;
Akt/AKT,: protein kinase B;
APNG,: Alkylpurine-DNA-N-glycosylase;
ARG1,: Arginase 1;
ATF3,: activating transcription factor 3;
BBB,: blood-brain barrier;
BBTB,: blood-brain tumor barrier;
BER,: base excision repair;
Bregs,: regulatory B Cells;
BTG2,: B-cell translocation gene 2;
CCAT2,: colon cancer associated transcript 2;
CD,: cluster of differentiation;
ceRNA,: competing endogenous RNA;
cfDNA,: cell-free DNA;
circRNA,: circular RNA;
CAF,: cancer-associated fibroblast;
circCABIN1,: Circular RNA CABIN1;
CAV1,: caveolin-1;
circ-HIPK3,: Circular RNA HIPK3;
circWDR62,: Circular RNA WDR62;
CL, classical subtype, CRISPR-Cas9,: Clustered Regularly Interspaced Short Palindromic Repeats-associated protein 9;
CSC,: cancer stem cell;
CSF,: cerebrospinal fluid;
CT,: computed tomography;
CTCs,: circulating tumor cells;
CTLA-4,: cytotoxic T-lymphocyte-associated protein 4;
ctDNA,: circulating tumor DNA;
ctRNA,: circulating tumor RNA;
Cx43,: connexin 43;
DCs,: dendritic cells;
DLL1,: Delta-like ligand 1;
DNM3,: dynamin 3;
DPTIP,: 2,6-Dimethoxy-4-(5-Phenyl-4-(Thiophen-2-yl)-1H-Imidazole-2-yl)Phenol;
DSB,: double-strand break;
EGFR,: epidermal growth factor receptor;
EGFRvIII,: epidermal growth factor receptor variant III;
EMT,: epithelial–mesenchymal transition;
ERBB,: Erb-b2 receptor tyrosine kinase;
ERK,: extracellular signal-regulated kinase;
ERRFI1,: ERBB receptor feedback inhibitor 1;
ESCRT,: endosomal sorting complex required for transport;
EVs,: extracellular vesicles;
EVA1,: Eva-1 homolog A;
FBXW7,: F-box and WD repeat domain containing 7;
FDA,: Food and Drug Administration;
FasL,: Fas ligand;
FUS,: fused in sarcoma;
GBex,: glioblastoma-derived exosomes;
GBM,: glioblastoma;
GMP,: Good Manufacturing Practice;
GRB14,: growth factor receptor-bound protein 14;
GSCs,: glioma stem-like cells;
GW4869,: Neutral Sphingomyelinase Inhibitor GW4869;
GBM-EVs,: glioblastoma-derived extracellular vesicles;
GDEs,: glioma-derived extracellular vesicles;
HEY1,: hes-related family bHLH transcription factor with YRPW motif 1;
HIF-1α,: Hypoxia-inducible factor 1 alpha;
HLA,: human leukocyte antigen;
hnRNPA2B1,: heterogeneous nuclear ribonucleoprotein A2/B1;
HOTAIR,: HOX Transcript Antisense RNA;
HSP,: heat shock protein;
IDH1,: isocitrate dehydrogenase 1;
IDO/IDO1,: indoleamine 2,3-dioxygenase;
IFN-γ,: interferon gamma;
IL,: interleukin;
JAK,: Janus kinase;
JNK,: c-Jun N-terminal kinase;
LGALS9,: galectin-9;
lncRNA,: long non-coding RNA;
LRRC4,: leucine-rich repeat containing 4;
LRP1,: LDL Receptor related protein 1;
LSPR,: localized surface plasmon resonance;
MCAM,: melanoma cell adhesion molecule;
MGMT,: O6-methylguanine-DNA methyltransferase;
Mcl-1,: myeloid cell leukemia 1;
miR/miRNA,: microRNA;
MCT1,: monocarboxylate transporter 1;
MIF,: macrophage migration inhibitory factor;
MALAT1,: metastasis-associated lung adenocarcinoma transcript 1;
MDR,: multidrug resistance;
MISEV,: Minimal Information for Studies of Extracellular Vesicles;
MAPK,: mitogen-activated protein kinase;
MDSCs,: myeloid-derived suppressor cells;
MMP9,: matrix metalloproteinase 9;
MATE1,: multidrug and toxin extrusion protein 1;
MES,: mesenchymal subtype;
MRI,: magnetic resonance imaging;
MSCs,: mesenchymal stem cells;
mTOR,: mammalian target of rapamycin;
MVBs,: multivesicular bodies;
NANOG,: Homeobox Transcription Factor NANOG;
ncRNA,: non-coding RNA;
NF-κB,: nuclear factor kappa B;
NK cells,: natural killer cells;
NRF2,: nuclear factor erythroid 2– related factor 2;
nSMase2,: neutral sphingomyelinase 2;
OLFML3,: olfactomedin like 3;
PD-1,: programmed cell death protein 1;
PDGFR,: platelet-derived growth factor receptor;
PDGFRα,: platelet-derived growth factor receptor Alpha;
PD-L1,: programmed death-ligand 1;
PKM2,: pyruvate kinase M2;
PLAU, plasminogen activator,: Urokinase;
PLAT,: tissue plasminogen activator;
PlGF,: placental growth factor;
PN,: proneural subtype;
PTEN,: phosphatase and tensin homolog;
PTRF,: polymerase i and transcript release factor;
RAC1,: ras-related C3 botulinum toxin substrate 1;
Rab27a/b,: ras-related protein Rab-27A/B;
RNAi,: RNA interference;
RORα,: Retinoic Acid Receptor-Related Orphan Receptor Alpha;
RRM1,: Ribonucleotide Reductase Catalytic Subunit M1;
sEVs,: small extracellular vesicles;
SBF2-AS1,: SET Binding Factor 2 Antisense RNA 1;
SHG-44,: human glioma cell line SHG-44;
SKI-II,: Sphingosine Kinase Inhibitor II;
SLC47A1,: solute carrier family 47 member 1;
SMIs,: small-molecule inhibitors;
SNAP-23,: synaptosome-associated protein 23;
SNARE,: Soluble NSF Attachment Protein Receptor;
SNHG15,: Small Nucleolar RNA Host Gene 15;
S1P,: sphingosine-1-phosphate;
STAT3,: signal transducer and activator of transcription 3;
TAMs,: tumor-associated macrophages;
TREM1,: triggering receptor expressed on myeloid cells 1;
TAP1,: transporter associated with antigen processing 1;
TME,: tumor microenvironment;
TCEAL7,: transcription elongation factor A like 7;
TMZ,: temozolomide;
TDEs,: tumor-derived extracellular vesicles;
TNF-α,: tumor necrosis factor alpha;
TGF-β,: transforming growth factor beta;
TP53,: tumor protein p53;
THBS1,: thrombospondin 1;
TRAIL,: TNF-related apoptosis-inducing ligand;
TIM-3,: T-cell immunoglobulin and mucin-domain containing-3;
Tregs,: regulatory T cells;
U87MG,: human glioblastoma cell line U87MG;
VEGF,: vascular endothelial growth factor;
VEGFA,: vascular endothelial growth factor A;
XRCC4,: X-ray repair cross-complementing 4;
ZIC5,: Zic family member 5;
ZIP4,: Zrt/Irt-like protein 4;
XRCC4,: X-ray repair cross-complementing 4;
ZEB1,: zinc finger E-box binding homeobox 1.
